# Supplementary material for: Placebo and nocebo in clinical practice: An online cross-sectional survey of healthcare professionals from European countries on views, practices and training needs
Source: PLoS One. 2026 Jan 14;21(1):e0338905. doi: 10.1371/journal.pone.0338905 (PMC12834211; doi:10.1371/journal.pone.0338905)
Supplement: S1 File — (DOCX) [file pone.0338905.s001.docx]

**Supplementary Materials**

**S1 CHERRIES checklist**. Checklist for Reporting Results of Internet E-Surveys (CHERRIES)

| ***Checklist Item*** | ***Explanation*** | ***Page Number*** |
| --- | --- | --- |
| Describe survey design | Describe target population, sample frame. Is the sample a convenience sample? (In “open” surveys this is most likely.) | 5 |
| IRB approval | Mention whether the study has been approved by an IRB. | 5 |
| Informed consent | Describe the informed consent process. Where were the participants told the length of time of the survey, which data were stored and where and for how long, who the investigator was, and the purpose of the study? | 5 |
| Data protection | If any personal information was collected or stored, describe what mechanisms were used to protect unauthorized access. | 5-6 |
| Development and testing | State how the survey was developed, including whether the usability and technical functionality of the electronic questionnaire had been tested before fielding the questionnaire. | 6-7 |
| Open survey versus closed survey | An “open survey” is a survey open for each visitor of a site, while a closed survey is only open to a sample which the investigator knows (password-protected survey). | 5-6 |
| Contact mode | Indicate whether or not the initial contact with the potential participants was made on the Internet. (Investigators may also send out questionnaires by mail and allow for Web-based data entry.) | 5-6 |
| Advertising the survey | How/where was the survey announced or advertised? Some examples are offline media (newspapers), or online (mailing lists – If yes, which ones?) or banner ads (Where were these banner ads posted and what did they look like?). It is important to know the wording of the announcement as it will heavily influence who chooses to participate. Ideally the survey announcement should be published as an appendix. | 5-6 |
| Web/E-mail | State the type of e-survey (eg, one posted on a Web site, or one sent out through e-mail). If it is an e-mail survey, were the responses entered manually into a database, or was there an automatic method for capturing responses? | 5-6 |
| Context | Describe the Web site (for mailing list/newsgroup) in which the survey was posted. What is the Web site about, who is visiting it, what are visitors normally looking for? Discuss to what degree the content of the Web site could pre-select the sample or influence the results. For example, a survey about vaccination on a anti-immunization Web site will have different results from a Web survey conducted on a government Web site | 5-6 |
| Mandatory/voluntary | Was it a mandatory survey to be filled in by every visitor who wanted to enter the Web site, or was it a voluntary survey? | 5 |
| Incentives | Were any incentives offered (eg, monetary, prizes, or non-monetary incentives such as an offer to provide the survey results)? | 5-6 |
| Time/Date | In what timeframe were the data collected? | 5 |
| Randomization of items or questionnaires | To prevent biases items can be randomized or alternated. | 6 |
| Adaptive questioning | Use adaptive questioning (certain items, or only conditionally displayed based on responses to other items) to reduce number and complexity of the questions. | 6 |
| Number of Items | What was the number of questionnaire items per page? The number of items is an important factor for the completion rate. | 6-7 |
| Number of screens (pages) | Over how many pages was the questionnaire distributed? The number of items is an important factor for the completion rate. | - |
| Completeness check | It is technically possible to do consistency or completeness checks before the questionnaire is submitted. Was this done, and if “yes”, how (usually JAVAScript)? An alternative is to check for completeness after the questionnaire has been submitted (and highlight mandatory items). If this has been done, it should be reported. All items should provide a non-response option such as “not applicable” or “rather not say”, and selection of one response option should be enforced. | 7 |
| Review step | State whether respondents were able to review and change their answers (eg, through a Back button or a Review step which displays a summary of the responses and asks the respondents if they are correct). | 6 |
| Unique site visitor | If you provide view rates or participation rates, you need to define how you determined a unique visitor. There are different techniques available, based on IP addresses or cookies or both. | 6 |
| View rate (Ratio of unique survey visitors/unique site visitors) | Requires counting unique visitors to the first page of the survey, divided by the number of unique site visitors (not page views!). It is not unusual to have view rates of less than 0.1 % if the survey is voluntary. | - |
| Participation rate (Ratio of unique visitors who agreed to participate/unique first survey page visitors) | Count the unique number of people who filled in the first survey page (or agreed to participate, for example by checking a checkbox), divided by visitors who visit the first page of the survey (or the informed consents page, if present). This can also be called “recruitment” rate. | - |
| Completion rate (Ratio of users who finished the survey/users who agreed to participate) | The number of people submitting the last questionnaire page, divided by the number of people who agreed to participate (or submitted the first survey page). This is only relevant if there is a separate “informed consent” page or if the survey goes over several pages. This is a measure for attrition. Note that “completion” can involve leaving questionnaire items blank. This is not a measure for how completely questionnaires were filled in. (If you need a measure for this, use the word “completeness rate”.) | 7, 9-25 |
| Cookies used | Indicate whether cookies were used to assign a unique user identifier to each client computer. If so, mention the page on which the cookie was set and read, and how long the cookie was valid. Were duplicate entries avoided by preventing users access to the survey twice; or were duplicate database entries having the same user ID eliminated before analysis? In the latter case, which entries were kept for analysis (eg, the first entry or the most recent)? | - |
| IP check | Indicate whether the IP address of the client computer was used to identify potential duplicate entries from the same user. If so, mention the period of time for which no two entries from the same IP address were allowed (eg, 24 hours). Were duplicate entries avoided by preventing users with the same IP address access to the survey twice; or were duplicate database entries having the same IP address within a given period of time eliminated before analysis? If the latter, which entries were kept for analysis (eg, the first entry or the most recent)? | - |
| Log file analysis | Indicate whether other techniques to analyze the log file for identification of multiple entries were used. If so, please describe. | - |
| Registration | In “closed” (non-open) surveys, users need to login first and it is easier to prevent duplicate entries from the same user. Describe how this was done. For example, was the survey never displayed a second time once the user had filled it in, or was the username stored together with the survey results and later eliminated? If the latter, which entries were kept for analysis (eg, the first entry or the most recent)? | n/a |
| Handling of incomplete questionnaires | Were only completed questionnaires analyzed? Were questionnaires which terminated early (where, for example, users did not go through all questionnaire pages) also analyzed? | 6-7 |
| Questionnaires submitted with an atypical timestamp | Some investigators may measure the time people needed to fill in a questionnaire and exclude questionnaires that were submitted too soon. Specify the timeframe that was used as a cut-off point, and describe how this point was determined. | - |
| Statistical correction | Indicate whether any methods such as weighting of items or propensity scores have been used to adjust for the non-representative sample; if so, please describe the methods. | 7-8 |

This checklist has been modified from Eysenbach G. Improving the quality of Web surveys: the Checklist for Reporting Results of Internet E-Surveys (CHERRIES). J Med Internet Res. 2004 Sep 29;6(3):e34 [erratum in J Med Internet Res. 2012; 14(1): e8.]. Article available at [https://www.jmir.org/2004/3/e34](https://www.jmir.org/2004/3/e34/)/; erratum available <https://www.jmir.org/2012/1/e8/>. Copyright ©Gunther Eysenbach. Originally published in the [Journal of Medical Internet](http://www.jmir.org) Research, 29.9.2004 and 04.01.2012.

This is an open-access article distributed under the terms of the Creative Commons Attribution License (<https://creativecommons.org/licenses/by/2.0/>), which permits unrestricted use, distribution, and reproduction in any medium, provided the original work, first published in the Journal of Medical Internet Research, is properly cited.

**S2** Results of the Pilot study to assess usability, technical functionality, acceptability, and refine question wording for clarity.

***Pilot Demographics***

| Age  33/45 (73.3%) | Mean (SD) | 43.8 (10.95) |
| --- | --- | --- |
| Gender  35/45 (77.8%) | Female  Male  Non-binary  Other  Rather not say | 18 (51.4%)  17 (48.6%)  0  0  0 |
| Country  35/45 (77.8%) | United Kingdom  Greece  Austria  Spain  France  Ireland  Italy  Portugal  Other  *Albania, Algeria, Croatia, Estonia, India, Iraq, Lithuania, Norway, Pakistan, Serbia, Sweden, Ukraine* | 5 (14.29%)  4 (11.43%)  3 (8.57%)  3 (8.57%)  2 (5.71%)  2 (5.71%)  2 (5.71%)  2 (5.71%)  12 (26.7%) |
| Profession  37/45 (82.2%) | Medical Professional  Physiotherapy  Psychology  Nursing  Occupational Therapy  Other  *Chiropractor, Dentist* | 19 (51.4%)  8 (21.6%)  6 (16.2%)  2 (5.4%)  0  2 (5.4%) |
| Healthcare Specialty  36/45 (80.0%) | General practice / Family Medicine  Surgery  Rheumatology  Cardiovascular  Mental health (including psychiatry)  Neurology  Pain management  Rehabilitation Medicine  Gastroenterology  Anesthesiology  Pneumology  Urology  Oncology  Orthopedic  Dermatology  Cardiology  Pediatric  Psychology  Paramedic  Pharmacy  Other (please specify)  *Orofacial pain, Clinical pharmacology, Palliative care, Physiotherapy, Internal Medicine* | 4 (11.1%)  1 (2.8%)  1 (2.8%)  1 (2.8%)  4 (11.1%)  1 (2.8%)  23 (63.9%)  4 (11.1%)  1 (2.8%)  8 (22.2%)  0  0  0  4 (11.1%)  0  0  2 (5.6%)  5 (13.9%)  0  0  6 (16.7%) |
| Years of clinical experience  35/45 (77.8%) | Mean (SD) | 16.7 (11.1) |
| Days per week working clinically  34/45 (75.6%) | 1  2  3  4  5  6  7  Currently not working clinically | 5 (14.7%)  2 (5.9%)  1 (2.9%)  5 (14.7%)  12 (35.3%)  4 (11.7%)  1 (2.9%)  4 (11.8%) |
| How many patients do you manage per week?  33/45 (73.3%) | Mean (SD) | 24.6 (21.7) |
| What setting do you work in?  34/45 (75.6%) | Private  Public  Academia  Industry  Government based  City  Town  Rural  Mixed: city/rural  Other  *Humanitarian surgical project* | 10 (29.4%)  18 (52.9%)  12 (35.3.%)  1 (2.9%)  3 (8.8%)  7 (20.6%)  2 (5.9%)  1 (2.9%)  1 (2.9%)  1 (2.9%) |
| In what setting is this based?  34/45 (75.6%) | Hospital  Outpatient speciality care  Primary care/general practice  Other  *Private, University, Pathology Institute* | 19 (55.9%)  9 (26.5%)  9 (26.5%)  3 (8.8%) |

***Pilot Feedback***

| **Comment** | **Solution** |
| --- | --- |
| **"Who can take part? Qualified clinicians": Does it include people still in post-graduate training? Just to clarify.** | Survey was updated to clarify that both qualified clinicians and post-graduate trainees can participate. |
| **Question 25: To what extent does the placebo effect contribute to the effects of: vitamins etc. Necessary?** | Question 25 was reviewed and deemed necessary for understanding placebo effects across different interventions. |
| **Ethical concern about the use of pure placebo without patient consent** | Questions were revised to address ethical concerns about using pure placebo without patient consent. |
| **Move demographic questions to the end to maximize data collection** | Demographic questions were moved to the end to maximize data collection and completion rates. |
| **Q26 - "How ethical is..." dependent on context; add open response.** | An open response field was added to question 26 to allow respondents to elaborate on context-dependent ethics. |
| **Q41- Clarify "observation of clinicians" as not being formal training** | Q41 was clarified to distinguish between observation of clinicians and formal training. |
| **Clarify if the survey refers to Placebo, Placebo Effect, and Nocebo Effect outside clinical trials or in research.** | Survey now specifies whether placebo-related questions pertain to clinical trials or research. |
| **Add a question on prescribing treatments expecting a placebo effect.** | A new question was added to explore prescribing treatments expecting a placebo effect. |
| **Present questions one by one and adjust slider for progress visibility.** | Survey presentation was adjusted to display questions one by one, and the slider was improved for better progress tracking. |
| **Align answers horizontally or vertically, not mixed.** | Answer alignment was standardized to horizontal for consistency throughout the survey. |
| **Clarify difference between "placebo effect" and "placebo treatment" throughout.** | The distinction between "placebo effect" and "placebo treatment" was clarified throughout the survey. |
| **Include ethics approval code.** | An ethics approval code field was included in the survey. |
| **Clarify if "country" refers to birth or practice location.** | Clarification was added to specify whether "country" refers to birth or practice location. |
| **Q17: Replace "500 characteristics" with more meaningful wording.** | The phrase "500 characteristics" was replaced with more meaningful wording. |
| **Q10: Include online consultations as an option.** | An option for online consultations was included in Q10. |

**S3** Full survey

**
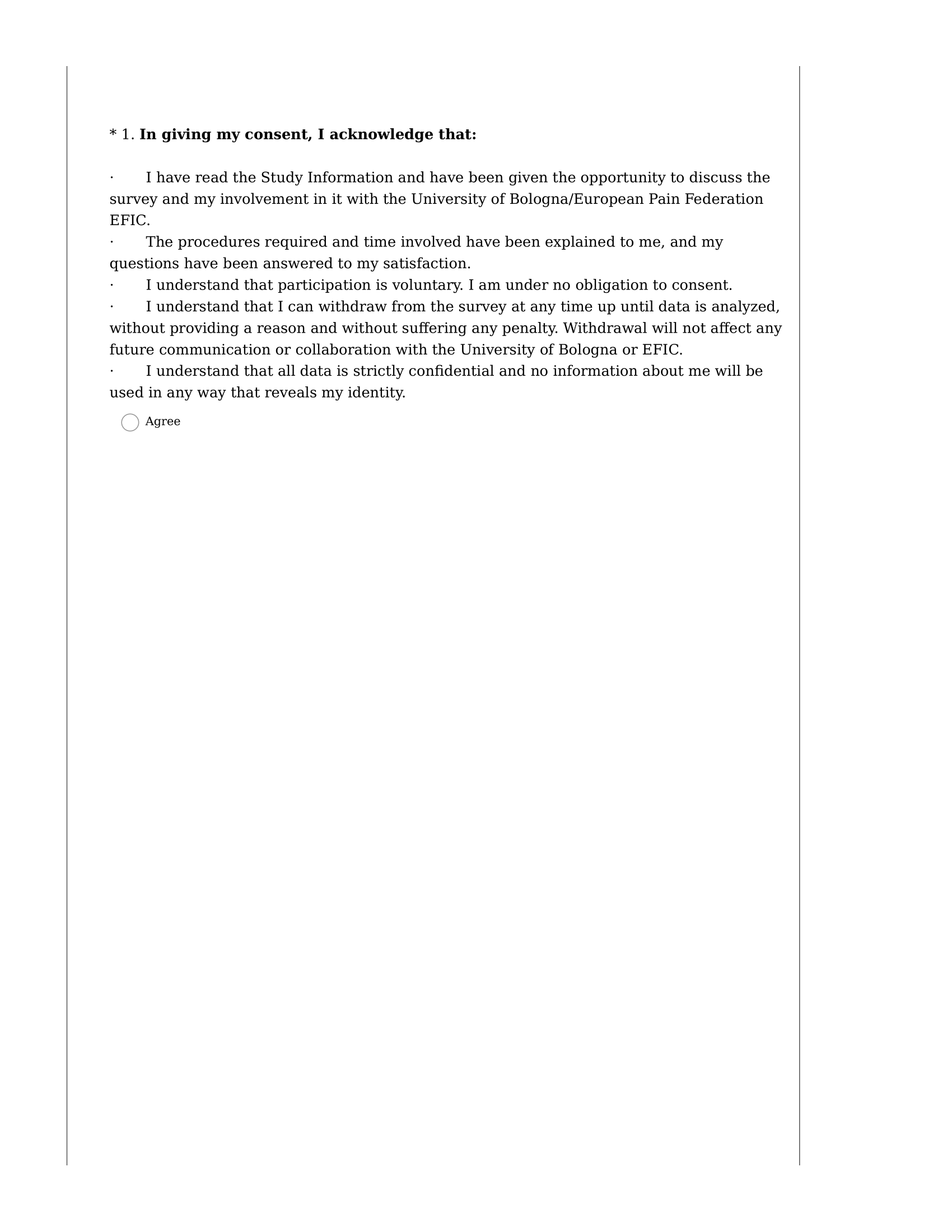

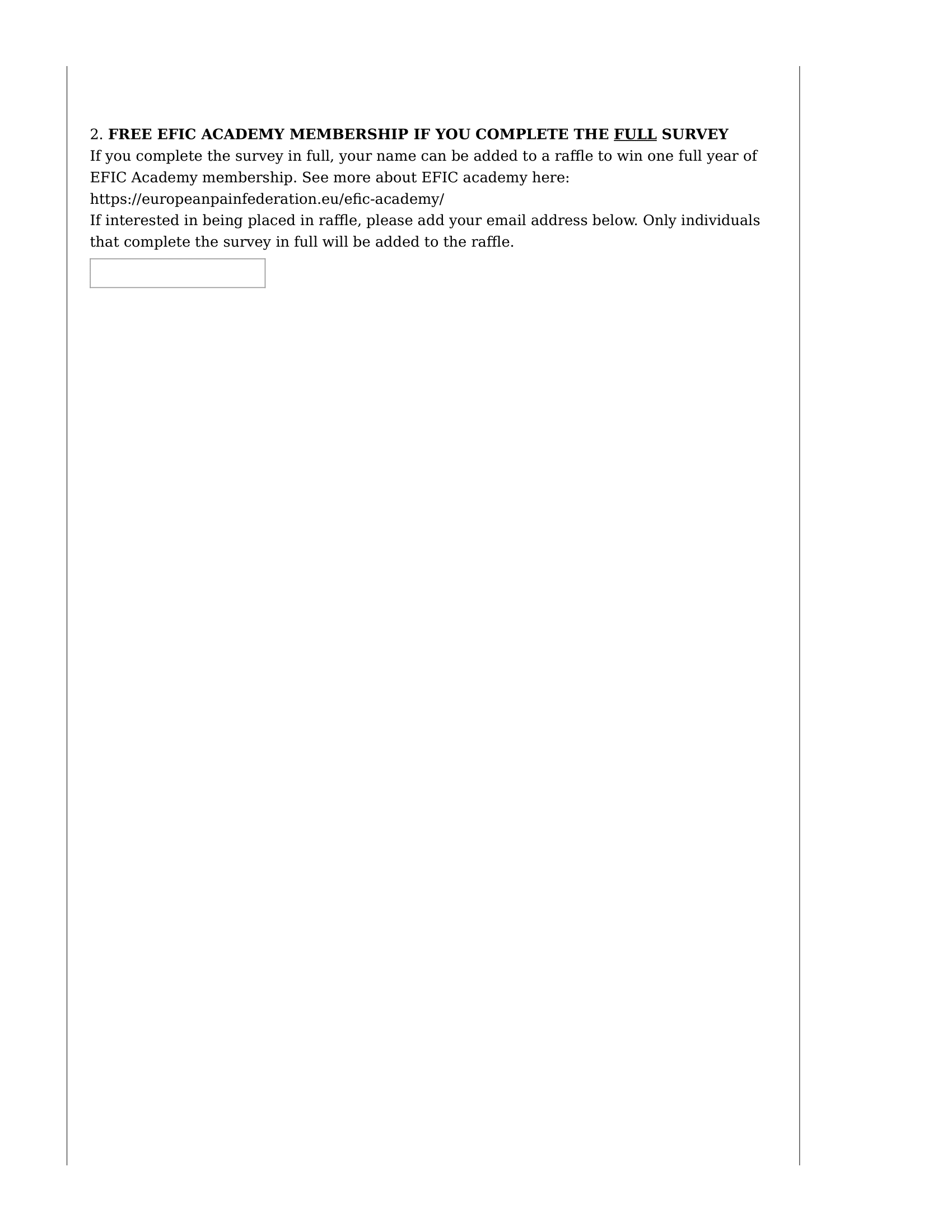

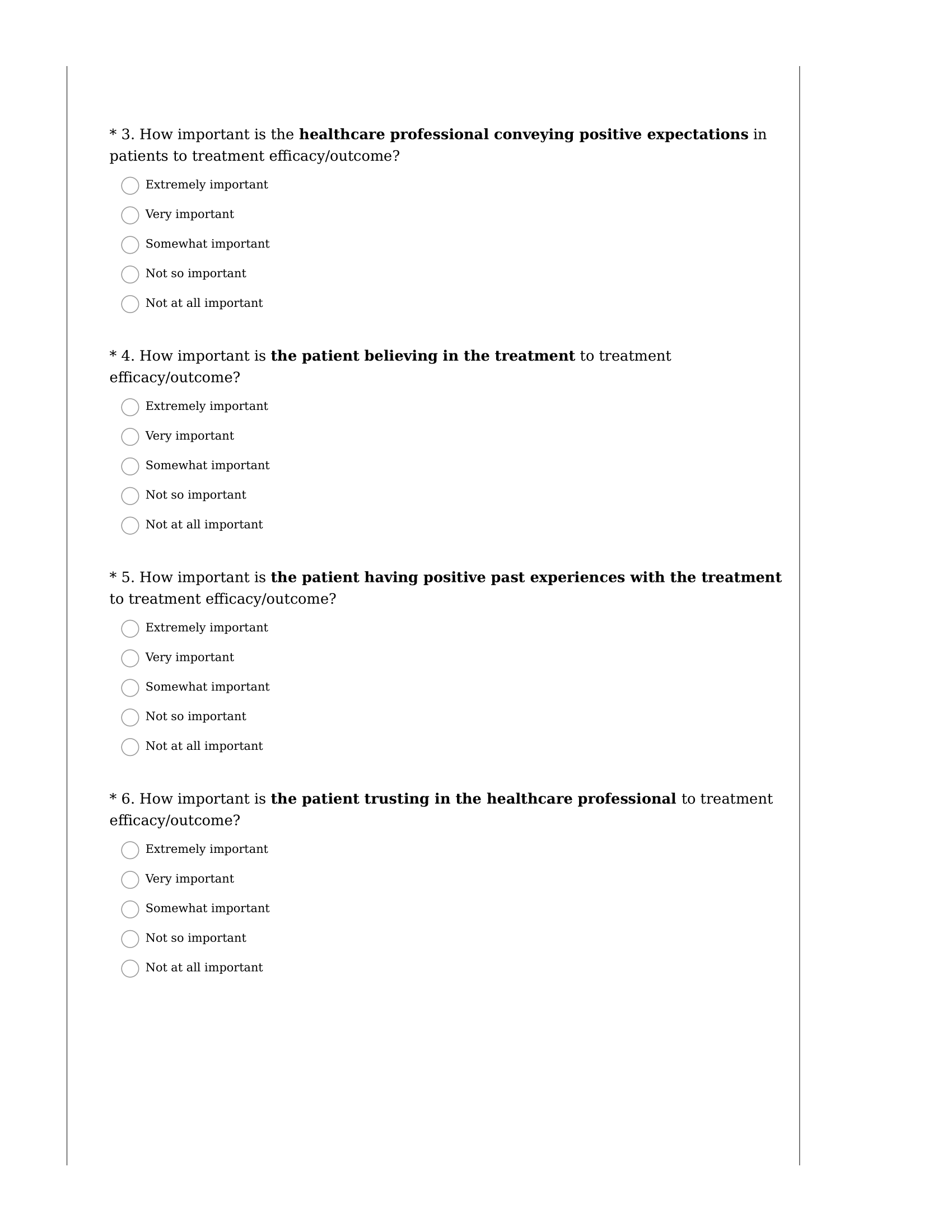

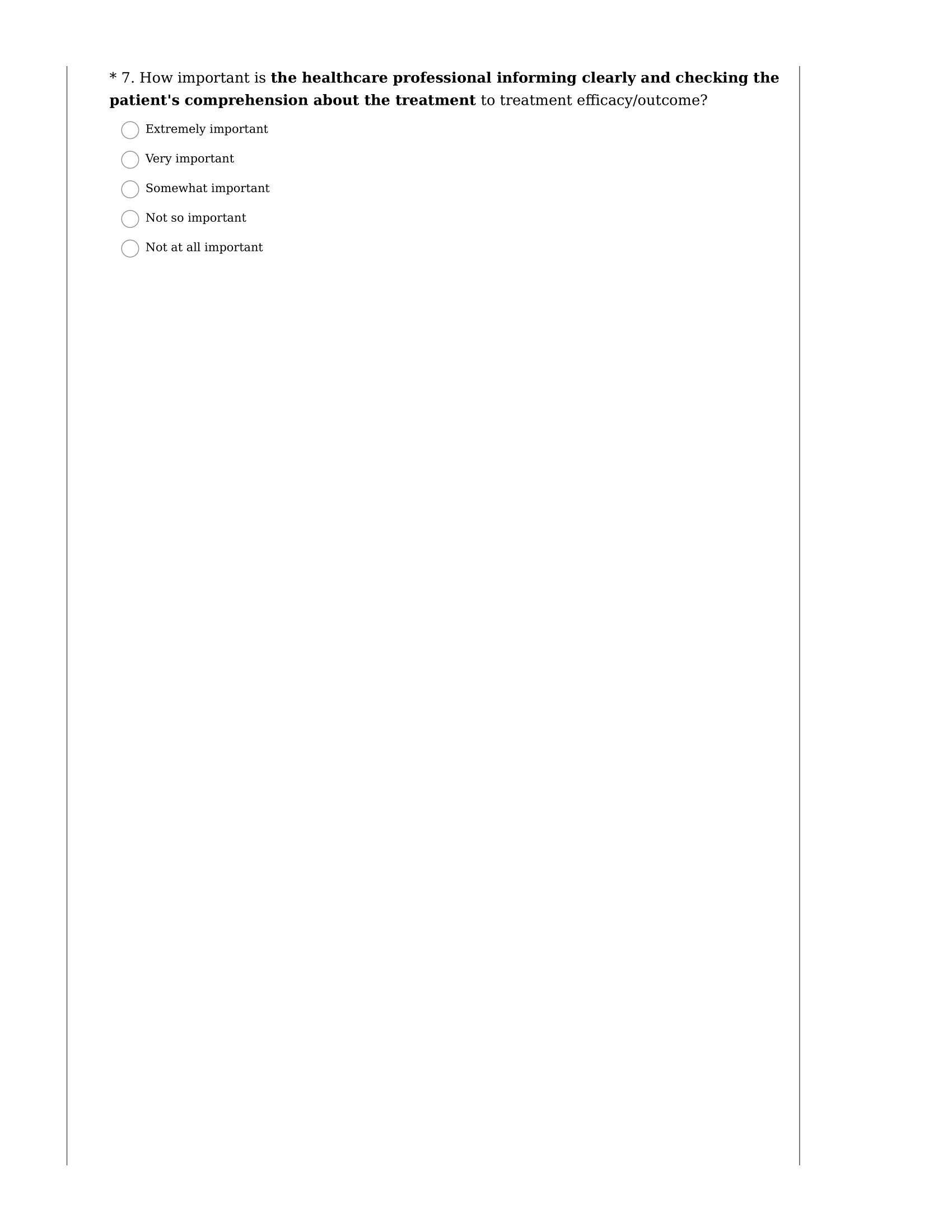

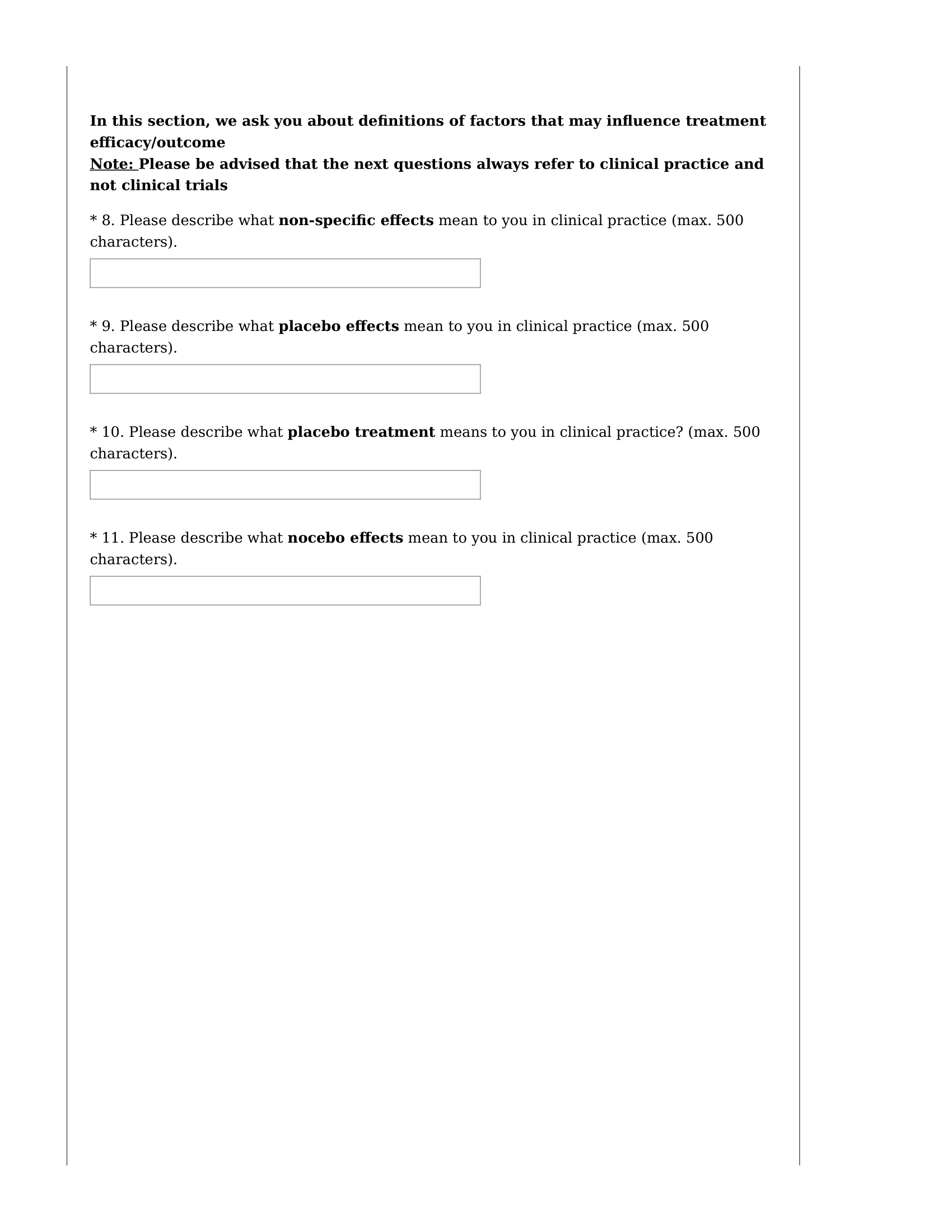

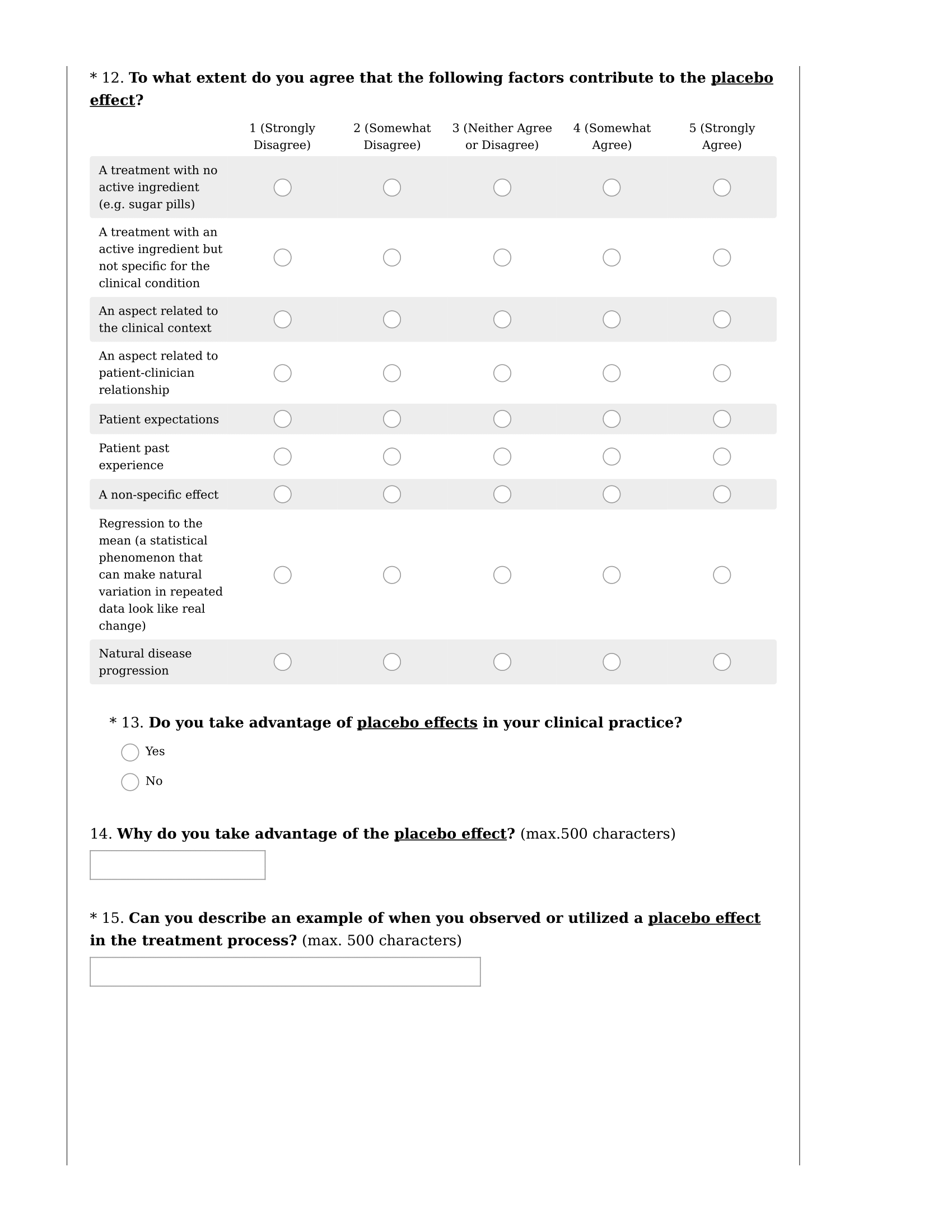

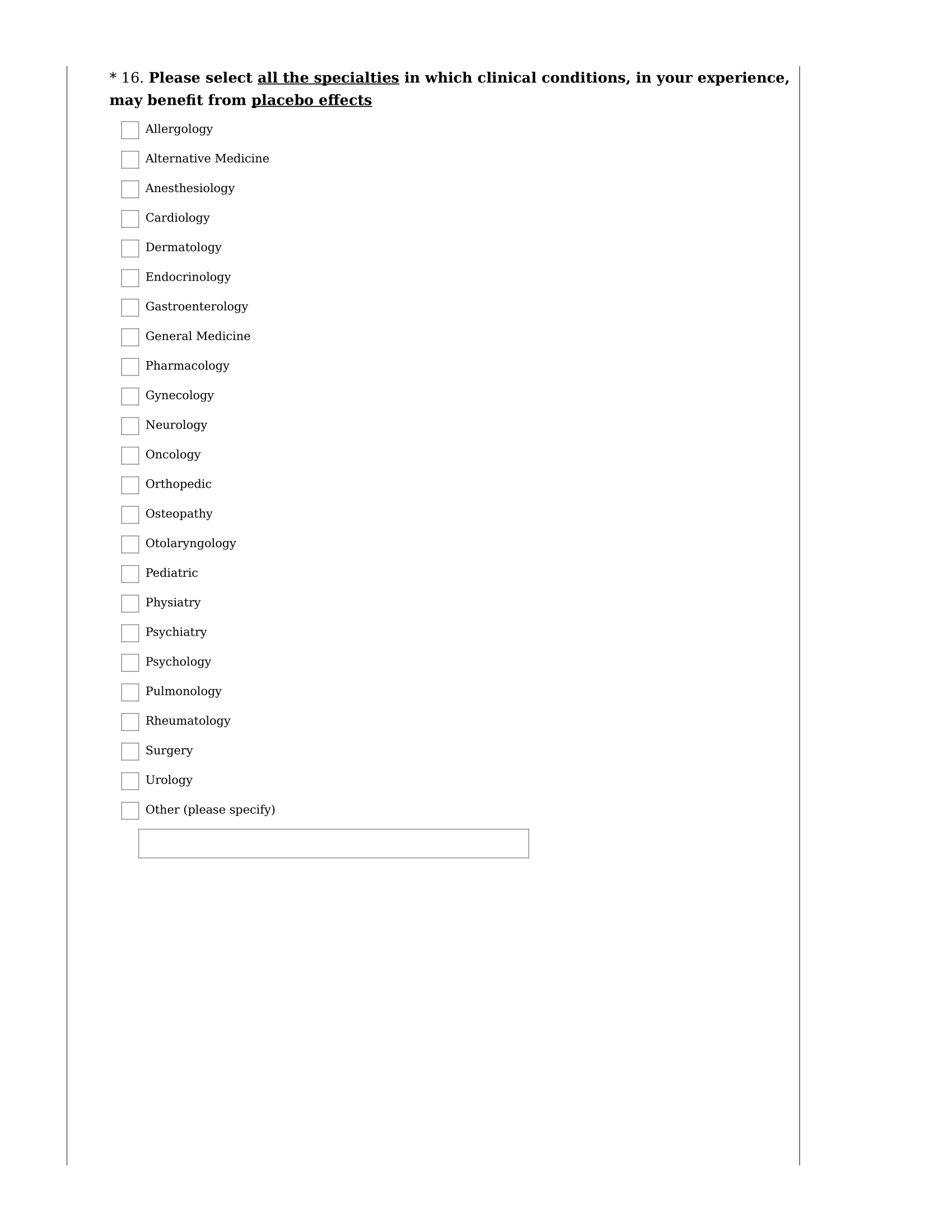

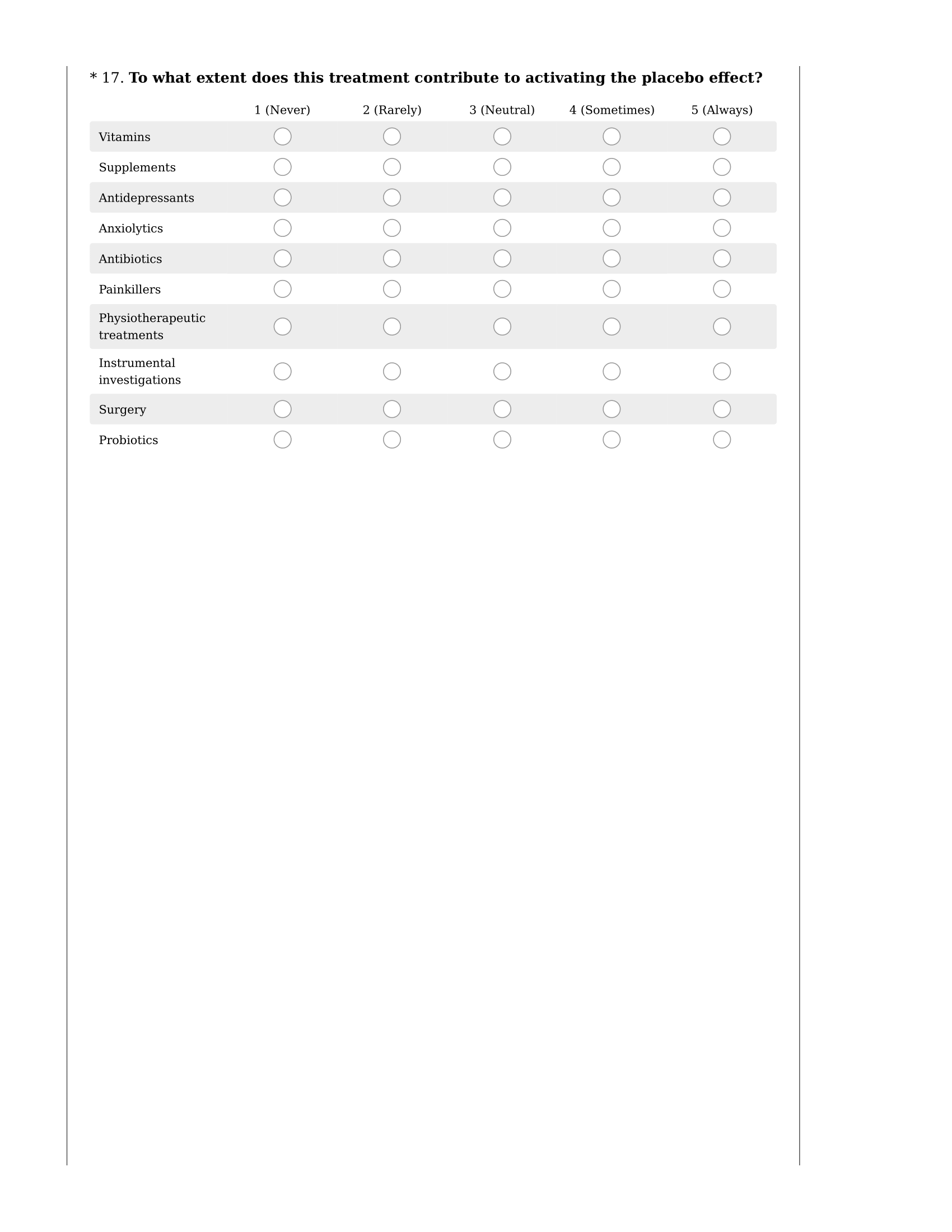

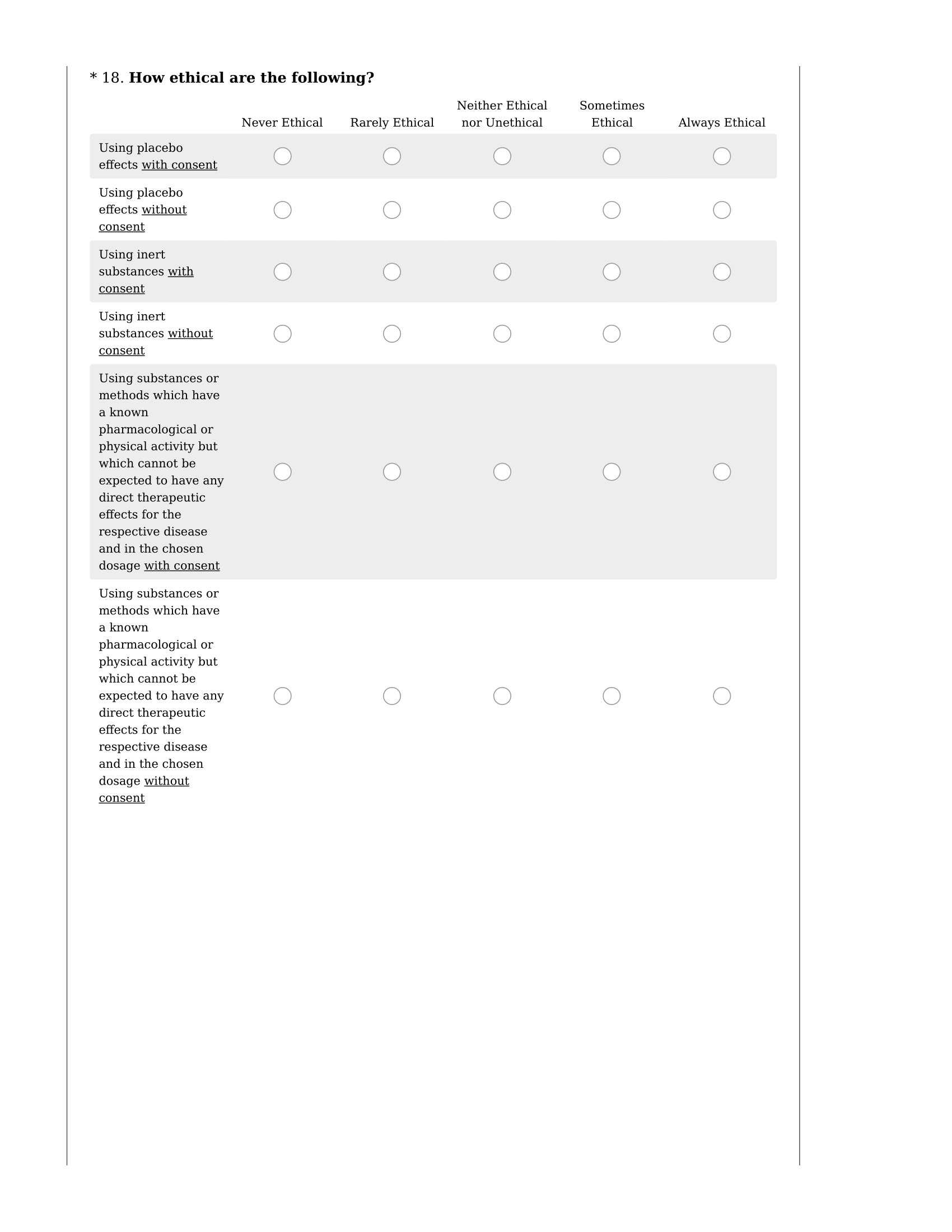

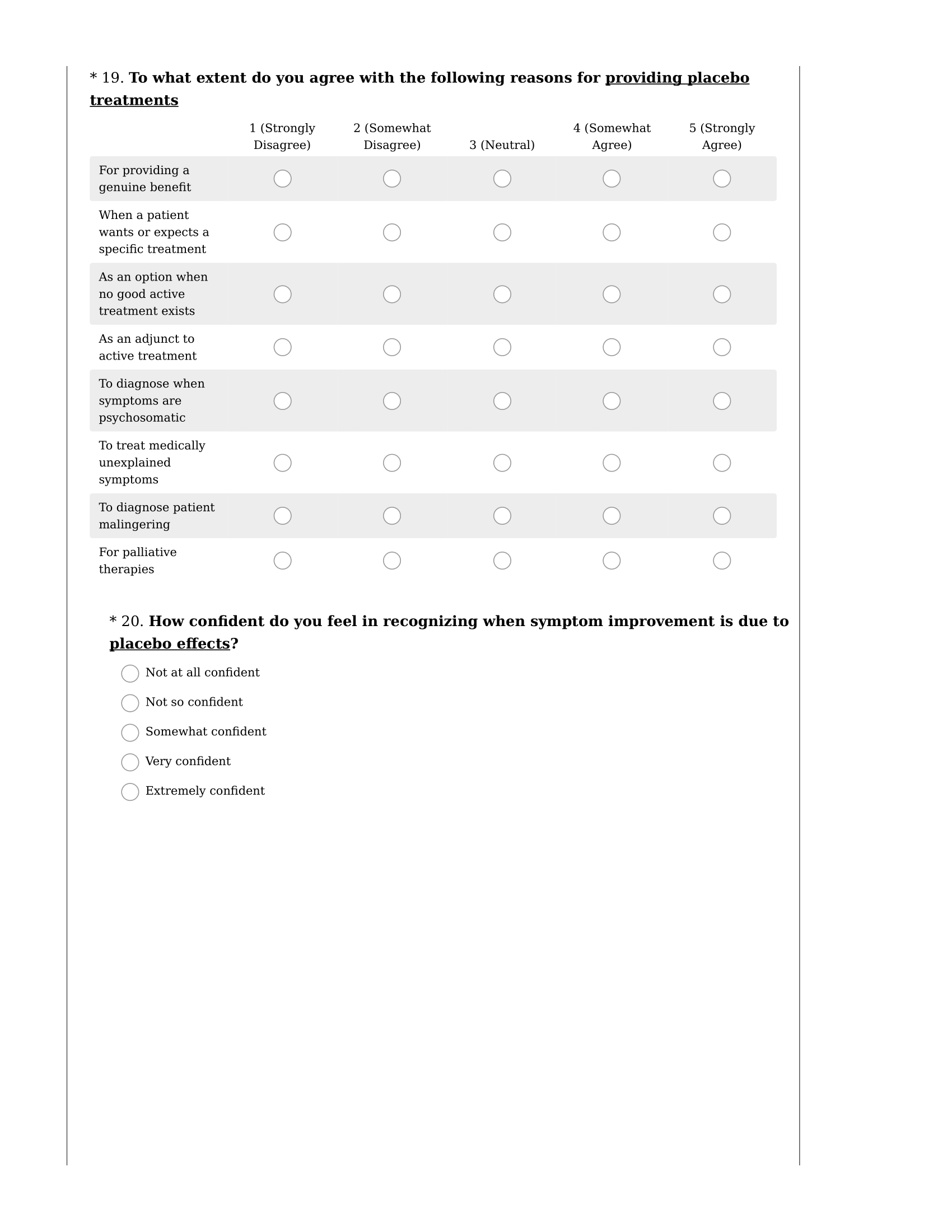

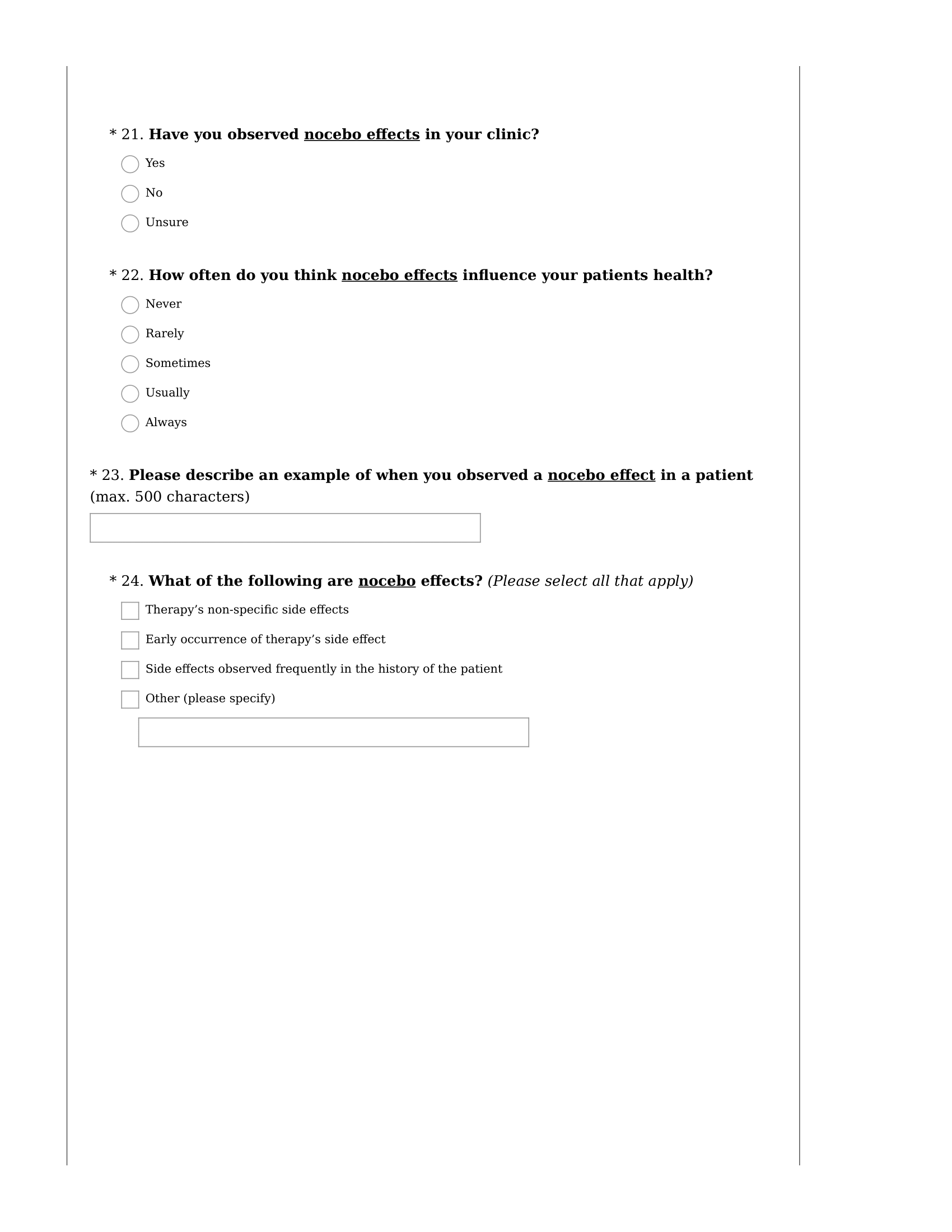

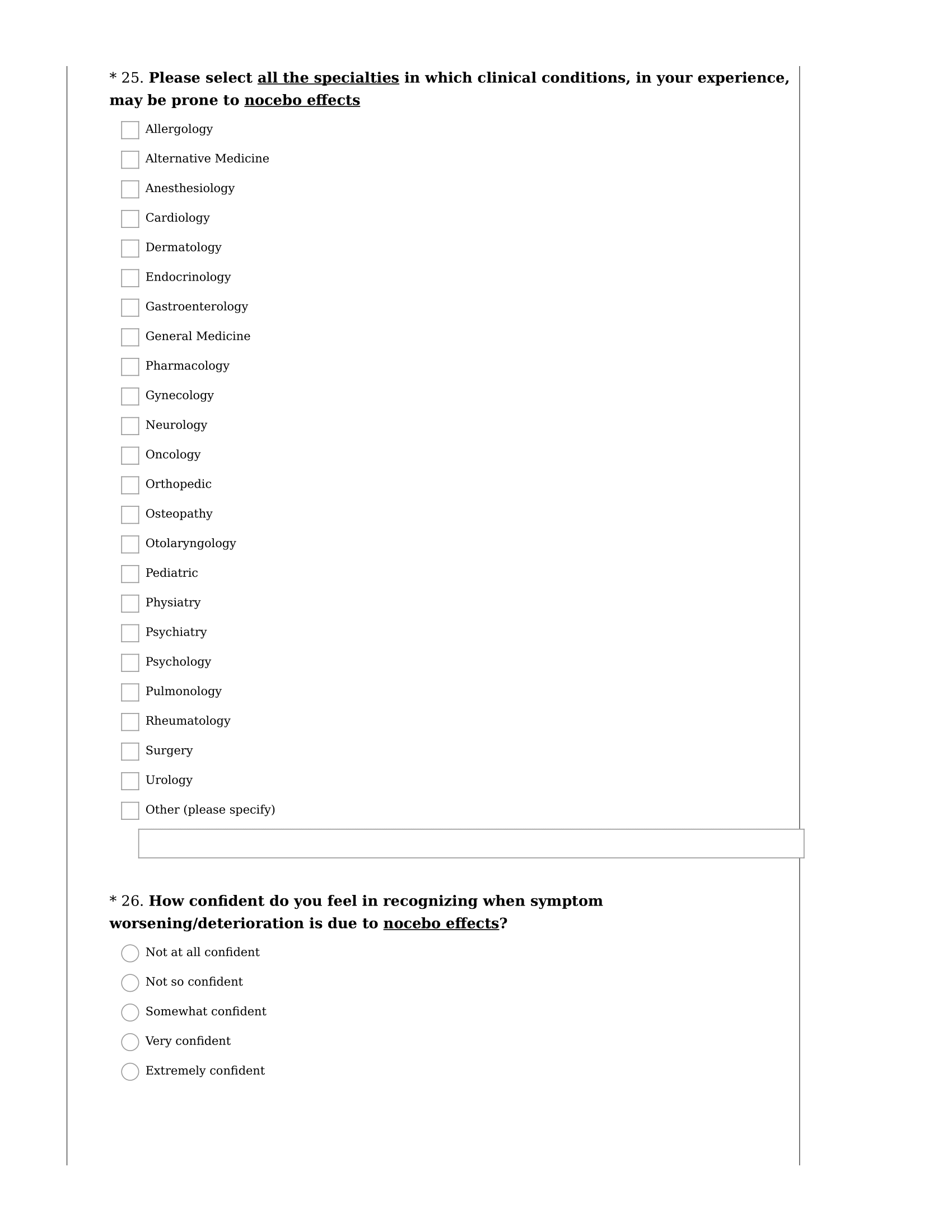

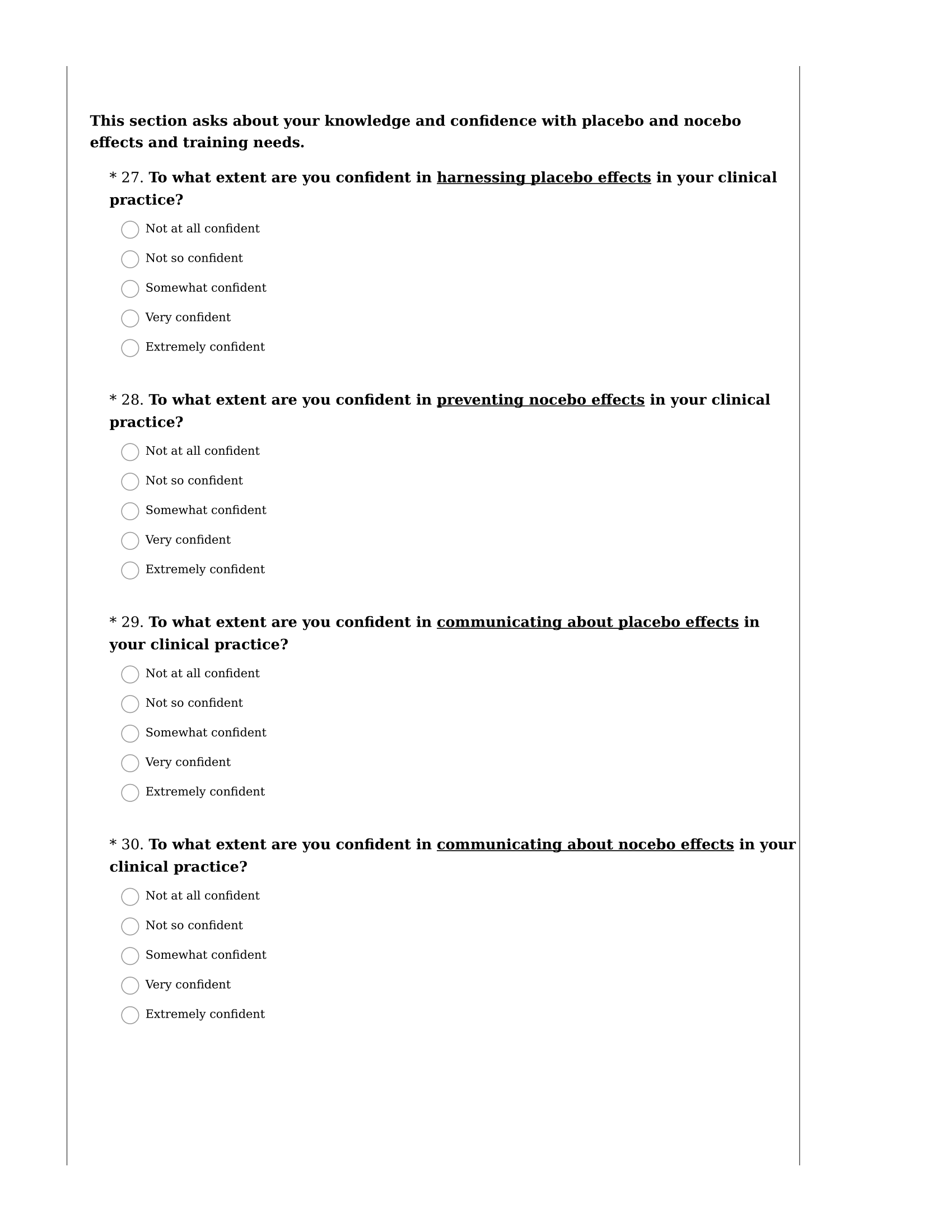

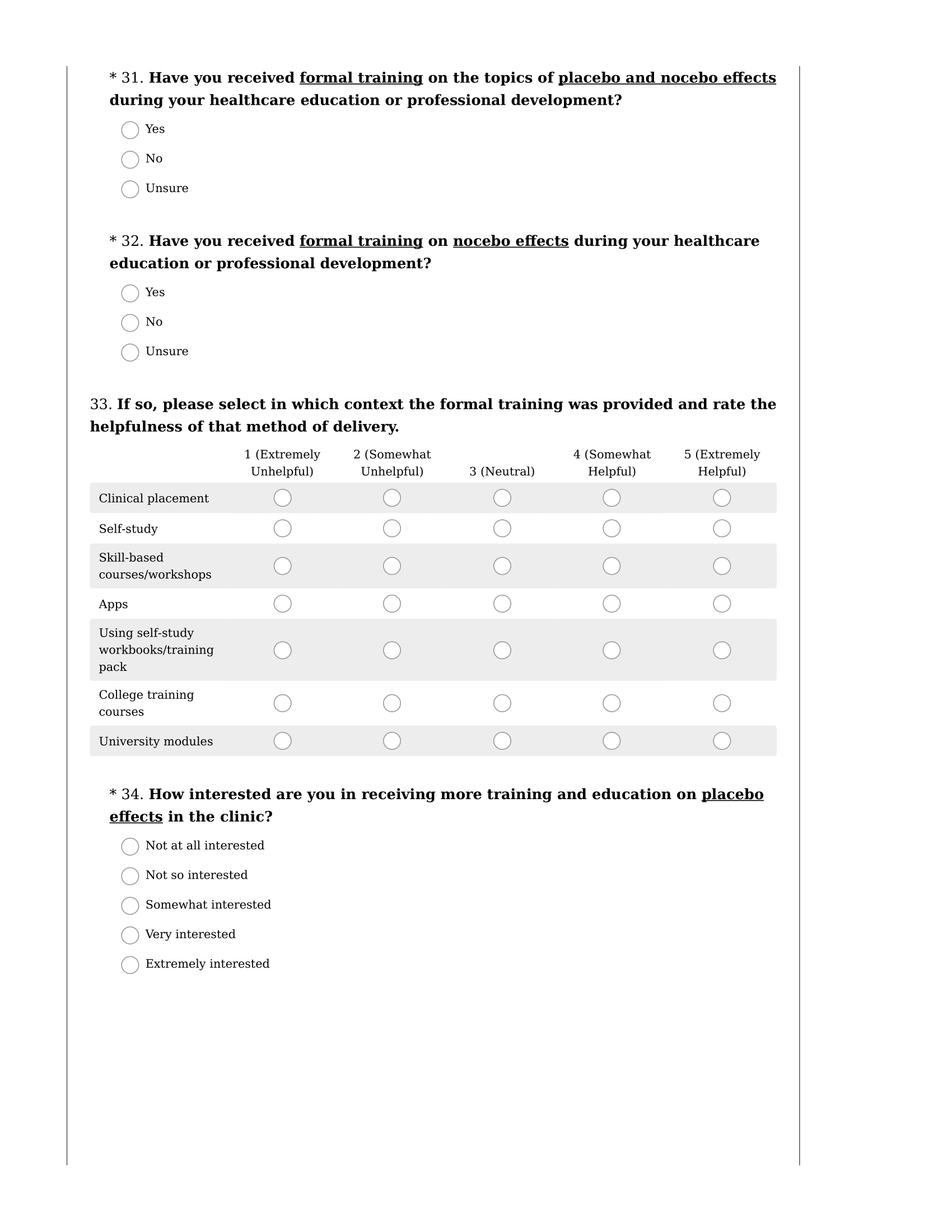

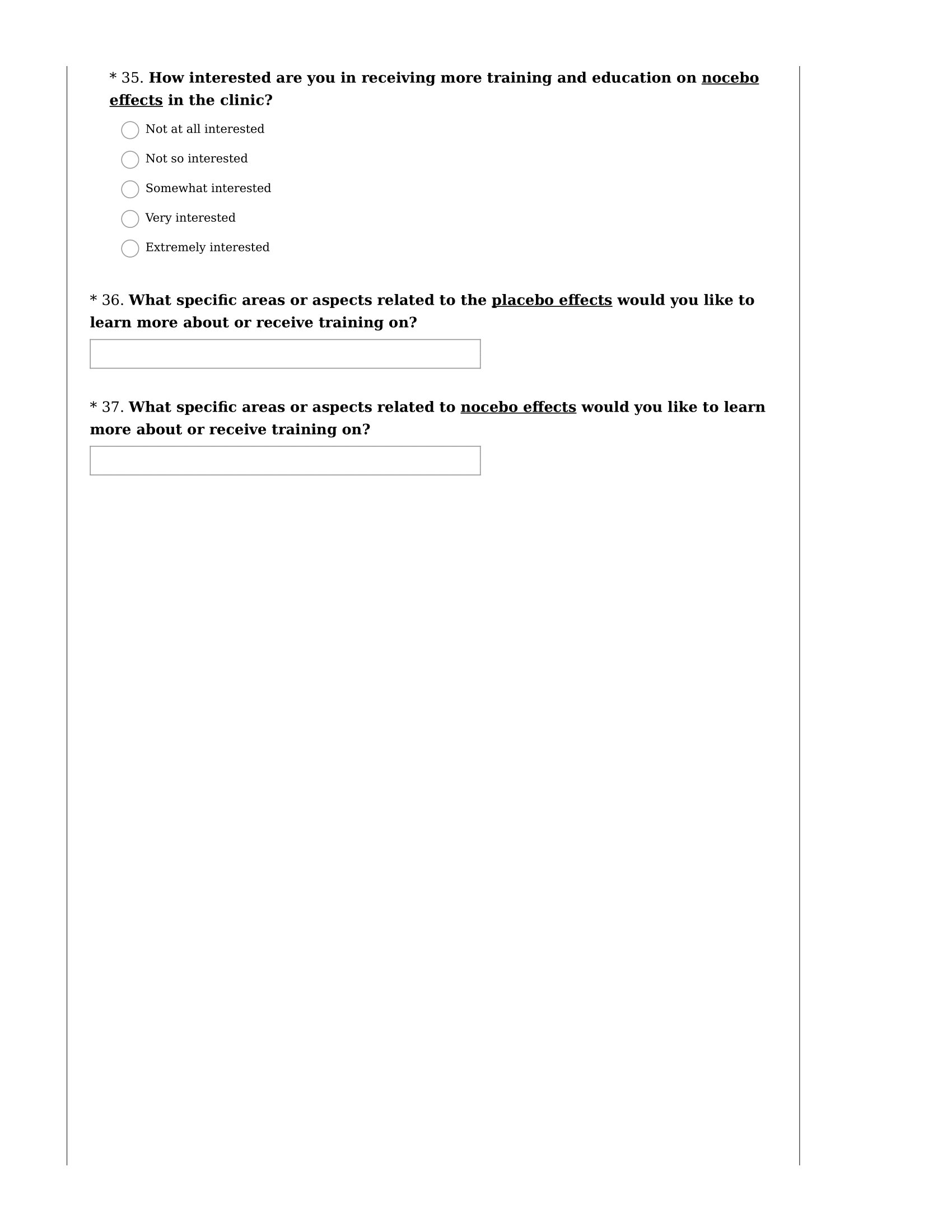

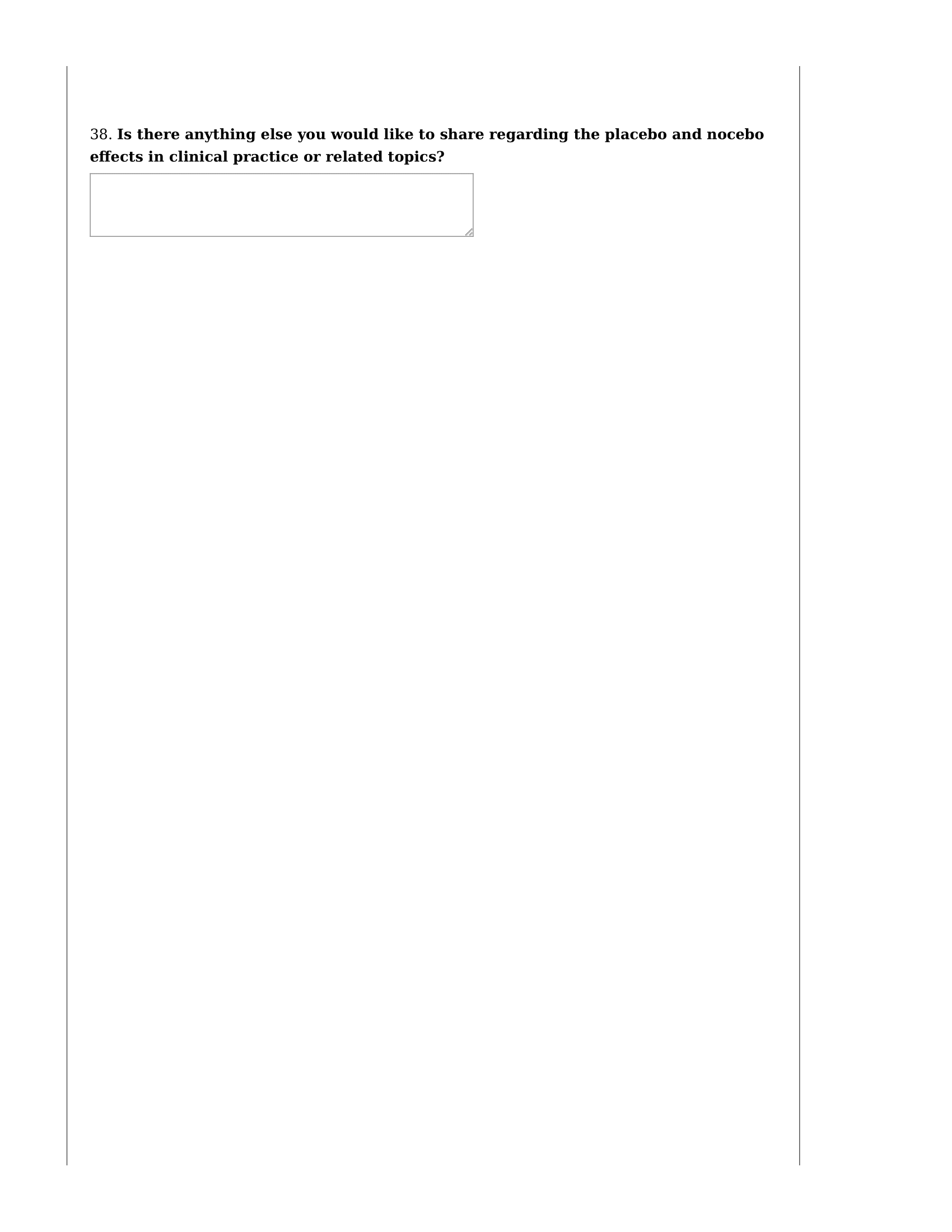

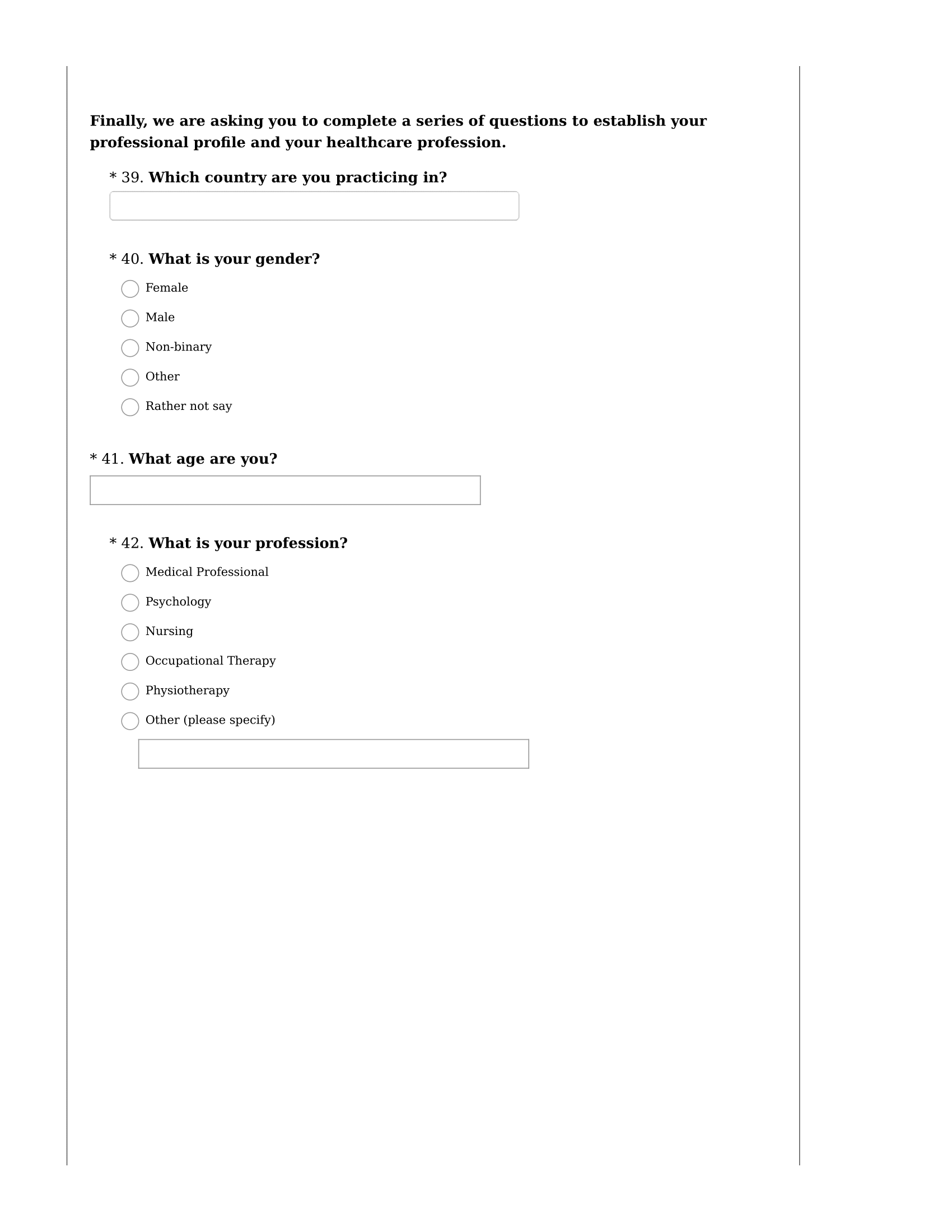

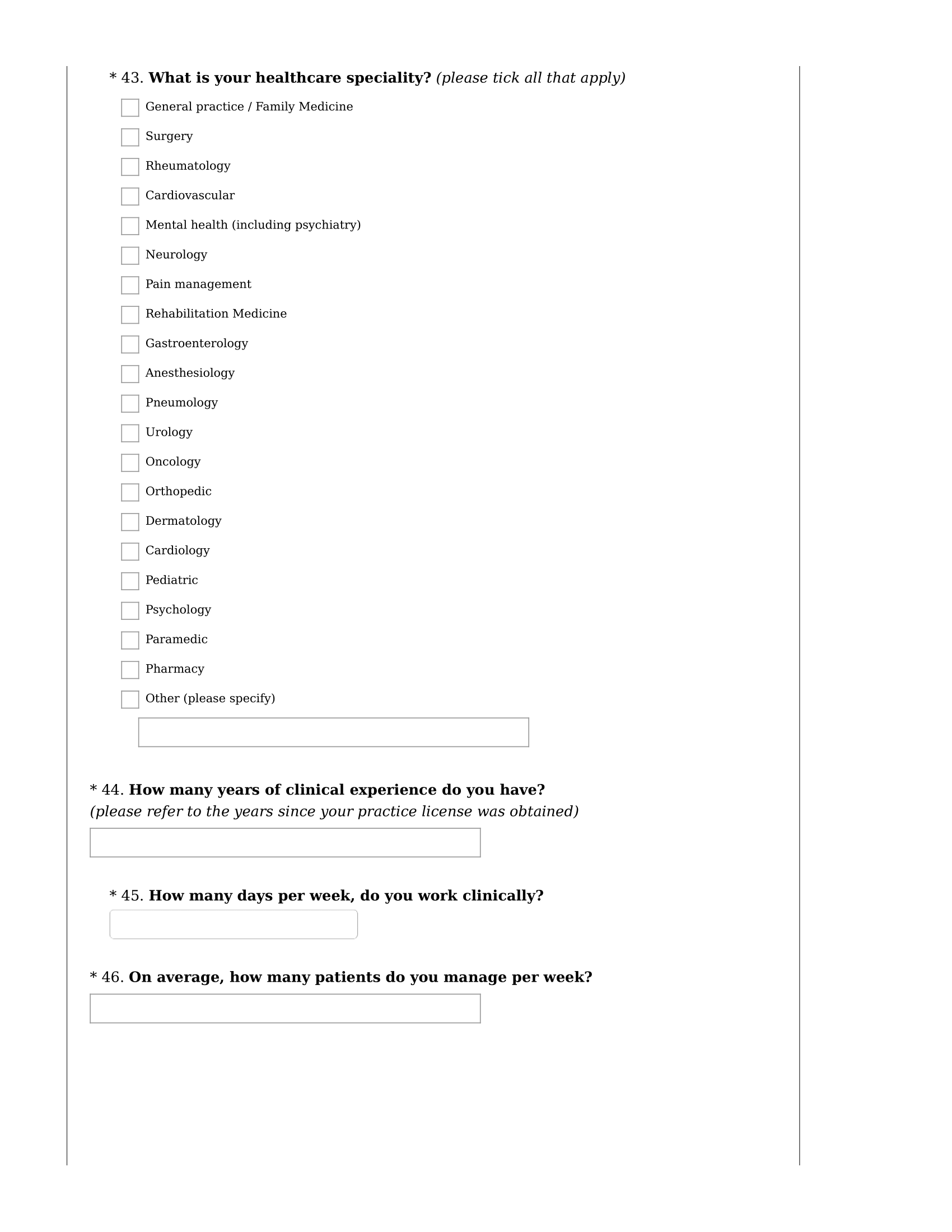

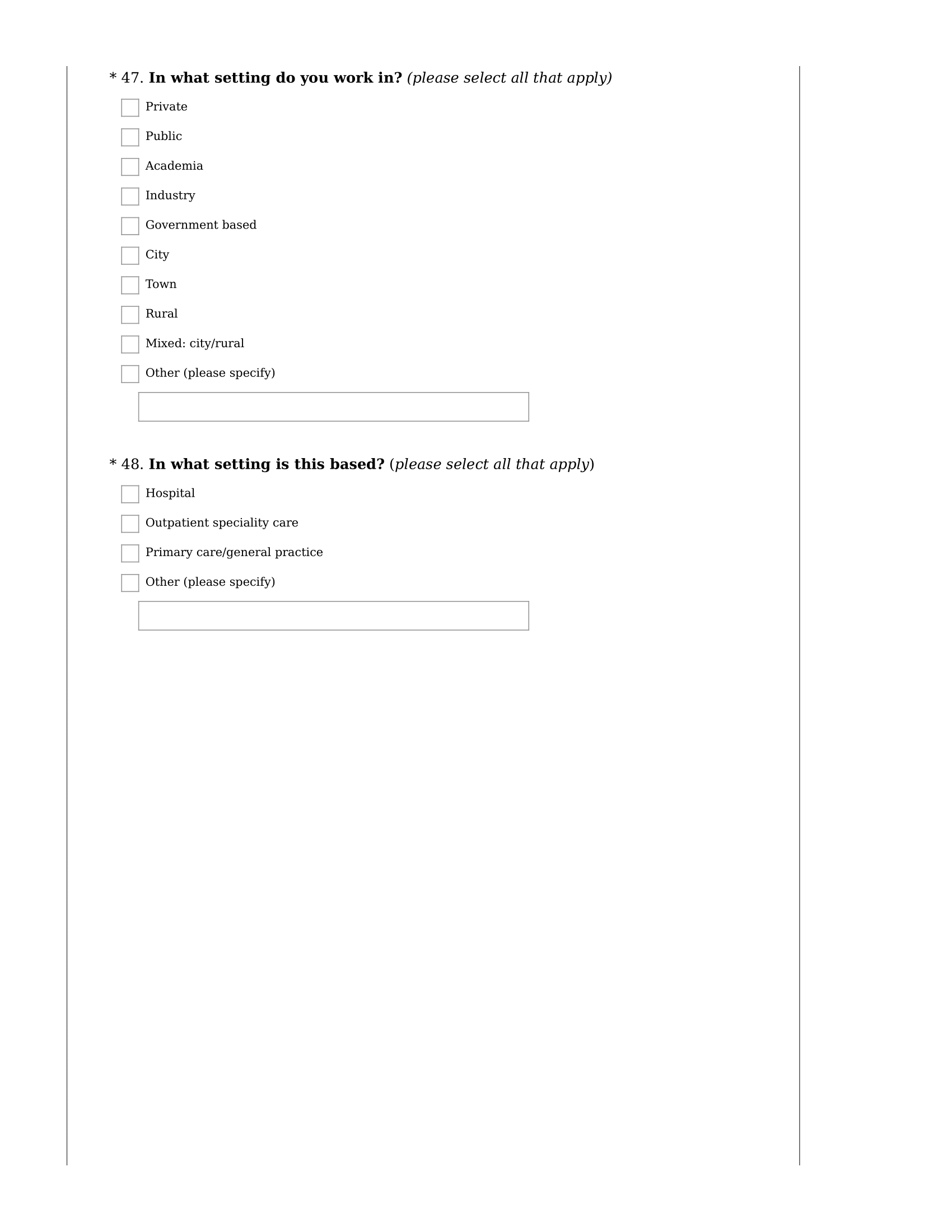

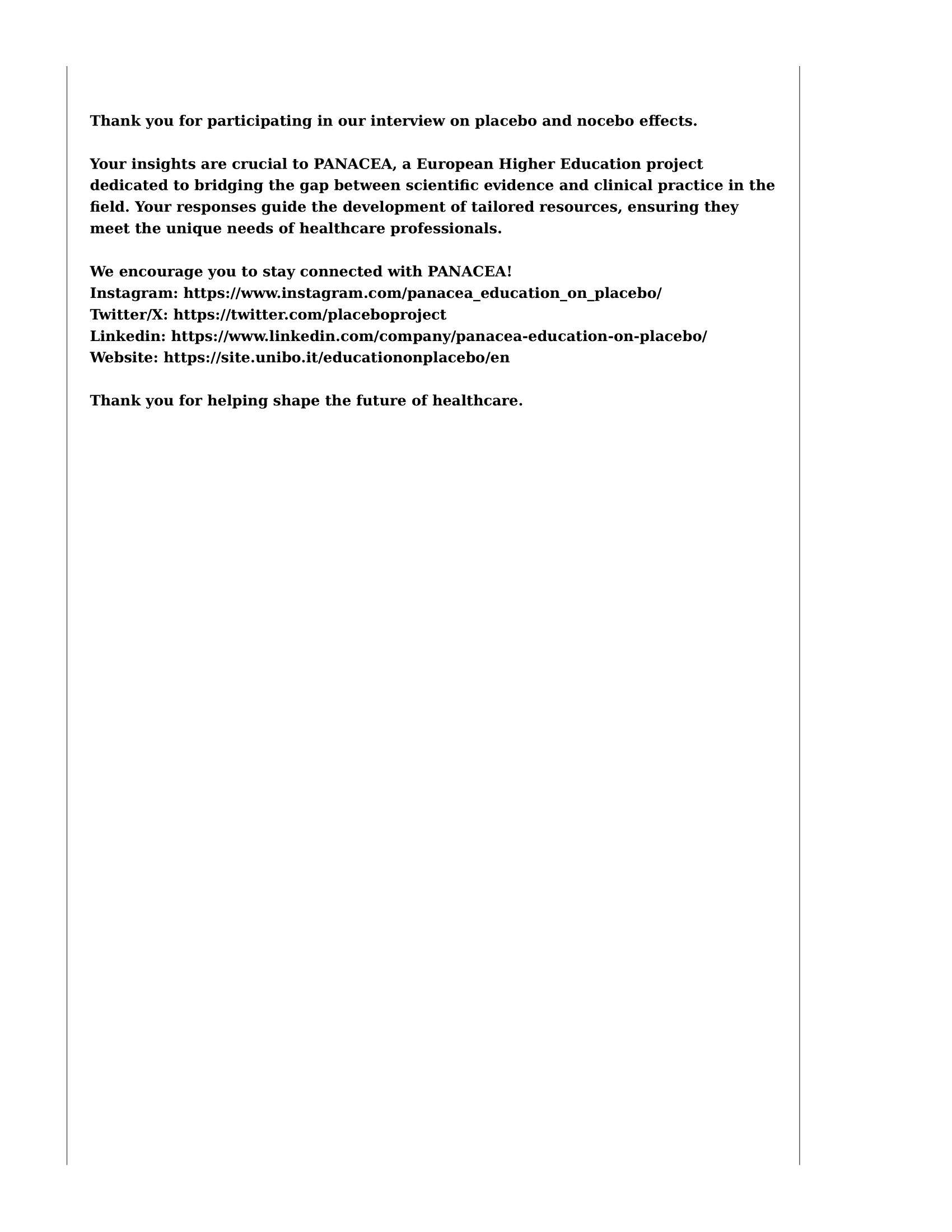
**

**S4** Additional examples of supporting quotes for the qualitative survey responses

| **Topic** | | **Examples of Supporting Quotes** |
| --- | --- | --- |
| **Knowledge and Understanding** | **Perceived Importance of Factors Influencing Patient Outcomes** | *“Effective communication from healthcare professionals sets the tone for the entire treatment process, shaping patient expectations and trust.”*  [Female, 40, Psychology] |
|  |  | *“Patient trust is foundational; without it, even the most advanced treatment may fail to yield results.”*  [Male, 55, Medical Professional] |
|  | **Definitions of Non-Specific Effects, Placebo, and Nocebo Phenomena** | *“Non-specific effects are those outcomes in patient health that are not directly due to the treatment itself but rather result from the context or the patient’s beliefs and experiences.”*  [Male, 38, Physiotherapy] |
|  |  | *“Placebo effects occur when patients experience improvement based on their belief in the treatment, not its inherent therapeutic properties.”*  [Female, 30, Psychology] |
|  |  | *“Nocebo effects are negative reactions that arise due to the patient’s negative expectations about a treatment.”*  [Male, 45, Medical Professional] |
|  | **Factors Contributing to Placebo and Nocebo Effects** | *“Patient expectations and past experiences play a major role; if they expect pain relief and have experienced positive outcomes before, the treatment is more likely to succeed.”*  [Female, 50, Medical Professional] |
|  |  | *“Negative framing and prior bad experiences can trigger nocebo effects even with harmless treatments.”*  [Male, 43, Psychology] |
|  | **Observation of Placebo and Nocebo Effects** | *“I’ve seen patients improve significantly when they trust the treatment and the healthcare provider, even if the treatment isn’t pharmacologically active.”*  [Female, 57, Nursing] |
|  |  | *“Patients sometimes report side effects that aren’t biologically plausible, which I attribute to nocebo responses.”*  [Male, 48, Medical Professional] |
|  | **Clinical Area Influenced by Placebo or Nocebo Effects** | *“Placebo effects are most evident in pain management and psychiatry, where the mental state and expectations play crucial roles.”*  [Male, 44, Pain Management] |
|  |  | *“I have noticed that nocebo effects are common in neurology, especially when discussing potential side effects with patients.”*  [Female, 39, Neurology] |
| **Applicability and impact in clinical practice** | **Healthcare Providers' Use of Placebo Effects** | *“In a clinical trial for a new pain medication, we incorporated a placebo group to assess the drug's efficacy. Interestingly, some participants in the placebo group reported reduced pain levels, highlighting how strong beliefs and perceptions can influence subjective experiences, even without active medication.”*  [Female, 39, Medical Professional] |
|  |  | *“When I give a supplement to help with a symptom and the main drug I administered seems to be not 100% effective. Most of the time I get a better feedback.”*  [Male, 45, Medical Professional] |
| **Training and Educational Needs** | **Areas of Interest for Further Training on Placebo Effects** | *“I would like to understand how to harness placebo effects in clinical practice, particularly through communication to enhance patient expectations and outcomes.”*  [Female, 35, Medical Professional] |
|  |  | *“Training on the ethical considerations and practical applications of placebo use would be invaluable.”*  [Male, 40, Physiotherapy] |
|  | **Areas of Interest for Further Training on Nocebo Effects** | *“Understanding how to minimize nocebo effects and prevent negative patient outcomes by addressing communication strategies and patient beliefs.”*  [Male, 50, Nursing] |
|  |  | *“Exploring the mechanisms behind nocebo effects and learning effective ways to communicate potential side effects without triggering nocebo responses would be beneficial.”*  [Female, 45, Psychology] |
