# Supplementary material for: Placebo and nocebo in clinical practice: An online cross-sectional survey of healthcare professionals from European countries on views, practices and training needs
Source: PLoS One. 2026 Jan 14;21(1):e0338905. doi: 10.1371/journal.pone.0338905 (PMC12834211; doi:10.1371/journal.pone.0338905)
Supplement: S3 File — (DOCX) [file pone.0338905.s003.docx]

Inclusivity in global research

PLOS’ policy on inclusivity in global research aims to improve transparency in the reporting of research performed outside of researchers’ own country or community and ensures that PLOS publications reporting global research adhere to high standards for research ethics and authorship. Authors of relevant research articles may be asked to complete the questionnaire below, which outlines ethical, cultural, and scientific considerations specific to inclusivity in global research. This questionnaire may be requested when researchers have travelled to a different country to conduct research, if research uses samples collected in another country, research with Indigenous populations or their lands, or if research is on cultural artefacts. Researchers travelling to another country solely to use laboratory equipment will not normally be required to complete the questionnaire. However, the questionnaire can be requested at the journal’s discretion for any submission – if you have been requested to complete this questionnaire by the PLOS journal you submitted to, please do so.

Please complete the questionnaire below and include this as a Supporting Information file with your manuscript. Note that if your paper is accepted for publication, this checklist will be published with your article in the supporting information files. Please ensure that you reference the checklist in the main body of your manuscript. We suggest adding a subsection ‘Inclusivity in global research’ to your Methods section and adding the following sentence: “Additional information regarding the ethical, cultural, and scientific considerations specific to inclusivity in global research is included in the Supporting Information (SX Checklist)”

The questions have been designed to be applicable to a wide range of study types, and there are subsections for both human subjects research and non-human subjects research. If any of the questions are not relevant to your research please mark them as “N/A” as appropriate.

**Ethical considerations, permits and authorship**

*This section is applicable to all research types.*

Provide details as to who granted permissions and/or consent for the study to take place in the Methods section of your manuscript. This should include the names of **all** ethics boards, governmental organizations, community leaders or other bodies that provided approval for the study. If individuals provided approval refer to these people by their role or title but do not list their name(s).

Reported on page number: 6

If there were any deviations from the study protocol after approval was obtained please provide details of these changes in the Methods section of your manuscript.
Did this study involve local collaborators that are residents of the country where the research was conducted or members of the community studied? If you do not have any authors from said communities, please provide an explanation for this below.

Reported on page number: N/A

This study did involve local collaborators. The author team includes researchers based in Europe (e.g., Italy, Poland, and other European countries), where the survey was conducted and the participant community is located. As the survey targeted healthcare professionals across Europe rather than a single country or community, the involvement of collaborators from multiple European institutions ensured local knowledge and contextual understanding were incorporated into study design, recruitment, and interpretation.

Everyone listed as an author should meet PLOS’ criteria for authorship and all individuals who meet these criteria should be included in the author byline, rather than the acknowledgements. For further information please see the journal’s Authorship Policy.

**Human subjects research (e.g. health research, medical research, cross-cultural psychology)**

Did you obtain written informed consent from a representative of the local community or region before the research took place? How did you establish who speaks for the community? Details of written informed consent obtained from study participants should be reported separately in the Methods section of your manuscript.

This study did not involve a specific local community requiring collective consent. The research was an online survey of healthcare professionals across multiple European countries, conducted with institutional ethical approval from the Bioethical Committee of the University of Bologna (protocol no. 0388068). Individual informed consent was obtained electronically from all participants before survey initiation, as described in the Ethics and Consent section of the manuscript.

How did members of the local community provide input on the aims of the research investigation, its methodology, and its anticipated outcome(s)?

Members of the local professional community provided input through the structure of the PANACEA Consortium, which includes collaborators across multiple European countries. The survey was developed with input from this consortium and the author team, drawing on their diverse expertise and perspectives. In addition, the questionnaire was pre-tested with 45 healthcare professionals from the EFIC mailing list to ensure usability, clarity, and relevance before launch. This process ensured that the aims, methodology, and anticipated outcomes reflected the needs and perspectives of the healthcare community being studied.

When engaging with the local community, how did you ensure that the informed consent documents and other materials could be understood by local stakeholders?

All participants were healthcare professionals, who were expected to have sufficient English proficiency to understand the survey and consent information. The consent statement was written in clear, accessible English and presented on the first page of the survey, with a mandatory checkbox confirming consent before proceeding. To further ensure clarity, the survey and consent materials were pre-tested with 45 healthcare professionals from the EFIC mailing list, and feedback was incorporated to refine the wording for usability and comprehension.

Will the findings of the research be made available in an understandable format to stakeholders in the community where the study was conducted (e.g. via a presentation, summary report, copies of publications, etc.)? Please provide details of how this will be achieved.

Yes. The findings will be made openly available through publication in PLOS ONE. In addition, results will be disseminated to stakeholders via the European Pain Federation EFIC’s communication channels (newsletter, website, social media) and through the PANACEA Consortium’s networks, which include clinicians, researchers, and professional bodies across Europe. Presentations at professional meetings such as the EFIC Congress will also ensure the results are shared in an understandable and accessible format with the healthcare community.

**Non-human subjects research using specimens/ animals collected as part of the study, or those housed in archival collections. Examples include archaeology, paleontology, botany and zoology.**

Did the permission you obtained from a local authority to perform the study include an agreement on access to outputs and benefit sharing? This may include procedures to enable fair distribution of the benefits and resources arising from the research performed. Please include any details of Prior Informed Consent and Benefit Sharing Agreements obtained. These may be required by field-specific regulations, for example the Convention on Biological Diversity (CBD) and the associated Nagoya Protocol.

N/A

If the material used in your study was imported, please A) provide the year it was imported and B) indicate whether permits were obtained to import/export the materials used, C) provide details of any permits obtained. If this information is not available, please indicate this.

N/A

If you used archival specimens, please state how the material used in your study was acquired by the institute it is held in and provide details of any permits obtained for the original excavations/ sample collection. If this information is not available, please indicate this.

N/A

How was the potential cultural significance of the materials collected in your study to local communities considered in your research design? Were Indigenous peoples and/or local researchers and institutions involved with archaeological excavations / collection of specimens? If so, please provide a description of their involvement.

N/A

If your manuscript includes photographs of human remains please indicate whether authors obtained permission from descendants or affiliated cultural communities to do so.

N/A
