# Supplementary material for: Placebo and nocebo in clinical practice: An online cross-sectional survey of healthcare professionals from European countries on views, practices and training needs
Source: PLoS One. 2026 Jan 14;21(1):e0338905. doi: 10.1371/journal.pone.0338905 (PMC12834211; doi:10.1371/journal.pone.0338905)
Supplement: Attachment — Submitted filename: review.pdf [file pone.0338905.s004.pdf]

PREAMBLE

Thank you for soliciting me to review this article entitled "*Placebo and Nocebo in Clinical Practice: An Online Cross-sectional Survey of European Healthcare Professionals Views, Practices and Training Needs*."

The following preamble is something I include in all my reviews: my intention is to assist you in strengthening your manuscript, and my comments are intended to be constructive. I understand (and have experienced) that the peer review process, often depersonalised, can be challenging, and commend you for your contributions to the field.

To this end, the comments are numbered in chronological order and color-coded as follows: major revisions are colored in red, minor revisions in orange, and suggestions in green. Please keep in mind that suggestions are just that: suggestions of personal opinion to improve the manuscript that the authors could take under consideration or not at their choosing, with no further justification. For ease of the process, line numbers are presented for each comment.

Please keep in mind that these comments are intended to enhance the overall quality of your work. I hope they'll help and will allow thoughtful consideration. I am happy to provide further clarification or discuss any aspect of this feedback. Expressing disagreement at this stage is beneficial to the reviewing process, especially when the arguments presented are constructive and sound.

Thank you for your commitment to advancing our understanding in this area, and I look forward to seeing the continued development of your research.

SUMMARY OF MANUSCRIPT REVIEW

This manuscript is clearly written and demonstrates a rigorously executed mixed-method study that adds depth to our understanding of healthcare professionals' knowledge and attitudes regarding placebo and nocebo effects. Particularly, I appreciated the correct use of descriptive statistics where incorrect use of p-values is widespread. While the study is promising and contributes valuable insights to the field, several minor revisions are needed to enhance clarity, accuracy, and consistency throughout the manuscript. I have also provided multiple suggestions for clarification and improvement. Particularly, the introduction would benefit from using more accurate references and deepening the rationale for the study's contribution. The results and discussion contain several ambiguities or inconsistencies that need to be addressed. In the discussion, overgeneralization from qualitative findings should be avoided, and several limitations should be acknowledged due to the sample size and composition.

Regards,

DETAILED MANUSCRIPT REVIEW:

MAJOR / MINOR / SUGGESTION

1. OVERALL: One thought that may be considered is to substitute the use of "the placebo effect" with "placebo effects" when appropriate. Because there are various mechanisms behind placebo effects and several levers to activate these mechanisms, it may be more accurate to evoke placebo effects in a plural sense.[1] Up to the authors.
2. INTRODUCTION: I believe a bibliometric reference would be better suited to support the claim on line 70 instead of references 4 & 5. Maybe something such as: Weimer, K., Buschhart, C., Broelz, E. K., Enck, P., & Horing, B. (2022). Bibliometric properties of placebo literature from the JIPS database: A descriptive study. Frontiers in Psychiatry, 13, 853953. https://doi.org/10.3389/fpsyt.2022.853953
3. INTRODUCTION: There may be some claims that the use of contextual factors does not produce clinically meaningful effects on their own. See:
    - Saueressig, T., Owen, P. J., Pedder, H., Arora, N. K., Simons, M., Kaczorowski, S., Miller, C. T., Donath, L., & Belavy, D. L. (2024). Boosting treatment outcomes via the patient-practitioner relationship, treatment-beliefs or therapeutic setting. A systematic review with meta-analysis of contextual effects in chronic musculoskeletal pain. The Journal of Orthopaedic and Sports Physical Therapy, 1–51. https://doi.org/10.2519/jospt.2024.12259
4. INTRODUCTION: I think the sentence on line 81-82 may need to be clarified.
5. INTRODUCTION: I'm not sure whether the question (L82) remains whether deceptive treatments can be administered ethically, but rather how placebo treatments may be used without infringing upon autonomy. To this end, open-label placebos have been a topic of growing interest. This is all the more relevant given that there are studies that have either found no superiority of a deceptive placebo over an open-label placebo[2], and one other that has demonstrated non-inferiority[3]. Nonetheless, deceptive placebo treatments are still largely used in practice.
6. INTRODUCTION: References 14 & 16 may not be the most suitable for the claim of line 86, given that these references were about placebo treatments and not implementing placebo effects in a larger sense (i.e., through the use of placebo treatments or the use of contextual factors).
7. INTRODUCTION: The references on line 98 mainly discuss acceptability from a physician's perspective. It could be worth adding one reference about the efficacy of

---

[1] "As emphasized in this review, we now know that there is not a single but many placebo effects, with different mechanisms and in different diseases, systems, and therapeutic interventions." – Benedetti, F., Carlino, E., & Pollo, A. (2011). How placebos change the patient's brain. Neuropsychopharmacology : Official Publication of the American College of Neuropsychopharmacology, 36(1), 339–354. https://doi.org/10.1038/npp.2010.81

[2] Locher, C., Frey Nascimento, A., Kirsch, I., Kossowsky, J., Meyer, A., & Gaab, J. (2017). Is the rationale more important than deception? A randomized controlled trial of open-label placebo analgesia. Pain, 158(12), 2320–2328. https://doi.org/10.1097/j.pain.0000000000001012

[3] Druart, L., Graham Longsworth, S. E., Terrisse, H., Locher, C., Blease, C., Rolland, C., & Pinsault, N. (2024). If only they knew! A non-inferiority randomized controlled trial comparing deceptive and open-label placebo in healthy individuals. European Journal of Pain, 28(3), 491–501. https://doi.org/10.1002/ejp.2204

OLPs. Additionally, the acceptability of OLPs seems to be somewhat different and more nuanced than that of physicians or surgeons. See (COI on one of these publications):

- Druart, L., Vauthrin, O., Pinsault, N., Locher, C., & Blease, C. (2023). 'It's not my greengrocer, it's someone from the medical profession': A qualitative study regarding acceptability of deceptive and open-label placebo prescribing in France. British Journal of Health Psychology, 28(2), 273–290. https://doi.org/10.1111/bjhp.12624
- Haas, J. W., Rief, W., & Doering, B. K. (2020). Open-label placebo treatment: Outcome expectations and general acceptance in the lay population. International Journal of Behavioral Medicine. https://doi.org/10.1007/s12529-020-09933-1
- Pan, Y., Frank, M. L., Kaptchuk, T. J., & Nestoriuc, Y. (2022). "Let's see what happens:"—Women's experiences of open-label placebo treatment for menopausal hot flushes in a randomized controlled trial. PLOS ONE, 17(11), e0276499. https://doi.org/10.1371/journal.pone.0276499

8. INTRODUCTION: On line 108, reference 22 should be mentioned. In that study, the population was exactly the target population mentioned: European healthcare professionals (HCP). Additionally, what difference does it make to have a survey for all HCPs? There are several surveys on physicians, medical students, nurses, nursing students, pharmacists, physiotherapists, and physiotherapy students. This might be worth expanding on in the following revision.
9. INTRODUCTION: The introduction could be strengthened (L106 onwards) with a greater emphasis on how this study contributes to the existing literature. As it stands, I'm unsure what this study brings to the existing body of literature. The main originality seems to be its focus on including nocebo effects and providing more in-depth information about the training HCPs might wish for.
10. METHODS: How was the survey developed (L127) regarding previous surveys? Were there sections that were taken verbatim? COSMIN recommendations for the use of measurement instruments are first to use existing ones and adapt only if necessary.
11. METHODS: Given the diverse population (L138), in how many languages was the questionnaire developed? How were the translations done? If only in English, I believe this would be a limitation of the study that should be mentioned. / It seems the answer comes on line 150.
12. METHODS: If the study is carried out in multiple different countries, would the ethical approval from an Italian ethics committee suffice? (L132) Would it be necessary to obtain ethical approval for each country? I don't think this is the case, but do the authors have any knowledge of this?
13. METHODS: How was healthcare professional defined in this context? (L138) From experience, there are different interpretations in the population about who is included as a HCP (e.g., osteopaths, chiropractors?), and variations exist among countries (e.g., psychologists). Were there any exclusion criteria?

14. METHODS: How do the authors explain the high rate of partially completed surveys? (L184) How far did the participants get into the survey before dropping out? This may be due to the questionnaire being too long. Recommendations are often to keep the questionnaire to a maximum of 7 minutes. What is the rationale for including the incomplete answers? How far did they go into the questionnaire? Also, I'm not sure I understand the fully and partially N=604 and N=807 figures. At the moment, I deduce that the 807 is the total number of consents, of which only 212 were complete and 604 were incomplete. So the N refers to the total number of questionnaires in one case and the number of incomplete questionnaires in another. Please clarify.
15. METHODS: I appreciate the appropriate use of descriptive statistics (L185) where the conditions for testing under H0 are not present. This is all the more worth highlighting given the pressure often put on researchers to produce p-values.
16. METHODS: Please provide a brief description of the profiles of the authors involved in the qualitative analysis (as suggested by the COREQ items). L193 onwards
17. METHODS: On line 204, why was the choice made to draw the framework from a theory-driven manner and not a data-driven one?
18. METHODS: Were the other authors then included in the qualitative analysis or was it solely the work of MOK and NS? L225
19. RESULTS: Was demographic data not included as a condition for the questionnaire to count as complete? If not, why was this choice made? Were the questions required or skippable? This is a very low portion of the respondents.
20. RESULTS: L244, with such a broad population base, the participation is very low. Several large countries were sparsely represented. This should be discussed as a limitation of the study's extrapolation to a larger population. The main participating countries are those of the most represented affiliation of authors. Perhaps in future studies, the PANACEA consortium should aim to establish a more extensive network throughout Europe. Notably, it is missing people/responses from Germany, the United Kingdom, France, and Spain, to name a few of the larger countries with active placebo researchers, in order of population.
21. FIGURE 1: What does the Other category comprise? This links to comment n°13
22. RESULTS: L254. Was there any mention of the other components of the placebo response apart from placebo effects?
23. RESULTS: I highly appreciate the direct inclusion of participant quotes in this presentation of the mixed data. It might be necessary to mention what the number is in quotes. I'm guessing it's the participant's age.
24. TABLE 1: I'm not clear on the options chosen for question 12. Are these questions about the contextual factors that trigger placebo or nocebo effects? If not, maybe consider switching from the word factor used in the question above to limit confusion. Additionally, an item such as "A treatment with an active ingredient" might have helped investigate whether participants understood that placebo responses and effects occur in every healthcare interaction.

25. RESULTS: I think the sentence line 308 should be rephrased, as conditions could also be a synonym of ailment or pathology. Here, maybe specialities, disciplines, or fields may be better suited.
26. TABLE 2: Why was it chosen to use different treatment instead of different forms of treatment? For instance, is it thought that pills have different placebo responses depending on whether they are given to someone with depression or anxiety? Wouldn't this be rather a confound with what conditions people think are susceptible to placebo responses, or even whether the treatments have a large specific effect, or if their effect is mainly a placebo response?
27. TABLE 2: In Table 1, the name of each subitem was given, and they are not in Table 2. Please pick one convention in reporting results.
28. TABLE 2: Were the placebo treatments described in the question? If the placebo treatment is pure or impure, deceptive or open, this may contextualize the responses.
29. RESULTS: This section should clarify whether it is referring to placebo treatments or enhancing placebo effects in daily care. This is correctly done in the table. On a side note, using placebo effects without consent could also occur without the knowledge of providers. In a way, HCPs who have little to no knowledge about placebo effects may still be promoting them intuitively. Additionally, I believe the second part of Table 2 is pertinent to the ethics of using placebo treatments and their effects.
30. TABLE 5: I think the title for Table 5 could be made more explicit. The text suggests that this was the result of asking how participants would like to learn about placebo and nocebo effects. The title of the table indicates that these are the preferred methods of learning for HCPs.
31. DISCUSSION: I'm not too fond of the term 'placebo intervention,' which may refer to a non-pharmacological placebo treatment (or placebo intervention), whereas I believe it was meant to say: high prevalence of the use of placebo treatments or effects. As the authors stated in the introduction, there is a need for crystal clear clarity on these terms and notions, thus my nitpicking.
32. DISCUSSION: I think "specialities" is more appropriate here (L399).
33. DISCUSSION: This finding of HCPs underestimating their role is similar to what was found on reference 22.
34. DISCUSSION: To my understanding, only placebo effect use was measured in this study (Question 13). Could you please check the use of 'placebo treatment' instead of 'effect' in this sentence, please?
35. DISCUSSION: I'm not sure the data here supports an inter-group cross-profession generalization (L411). It seems an over-simplification of rich qualitative data at first glance. At best, the qualitative data allows for the generation of the hypothesis that understanding varies across disciplines. These differences could also be due to age or area of practice. However, the analysis chose to look at profession, which could be the explanatory factor for these differences.

36. DISCUSSION: As a side note, it would be interesting to see if they felt more confident than others in harnessing placebo effects, given that they were prescribed as impure placebos by other professionals. L422
37. DISCUSSION: Was the lack of training mediated by years since graduation? This might help understand whether training is more implemented in initial health training or not. L439
38. DISCUSSION: These seem like findings rather than discussion points. They are probably the most relevant finding for this section of the survey. L447
39. DISCUSSION: The over-representation of pain professionals should be expected given the recruitment through the European Pain Federation EFIC. In addition to previous comments on the participants, it should be noted not only the overrepresentation but also the underrepresentation of specific demographics, as stated above. Thus, the first strength may be overstated.
40. DISCUSSION: I believe the ethicality could have benefited from a qualitative section as well. As it stands, I'm unsure it's more detailed than other surveys. In my opinion, the use of mixed methods here is the key strength of this project, and it allowed for an in-depth understanding of placebo knowledge that was lacking in several previous studies on the topic.
