# Supplementary material for: Placebo and nocebo in clinical practice: An online cross-sectional survey of healthcare professionals from European countries on views, practices and training needs
Source: PLoS One. 2026 Jan 14;21(1):e0338905. doi: 10.1371/journal.pone.0338905 (PMC12834211; doi:10.1371/journal.pone.0338905)
Supplement: Attachment — Submitted filename: Response to Reviewers R1.docx [file pone.0338905.s006.docx]

**Response to Reviewers**

PLOS ONE

**PONE-D-25-28687**

Placebo and Nocebo in Clinical Practice: An Online Cross-sectional Survey of European Healthcare Professionals Views, Practices and Training Needs

**Journal requirements**

1. Please ensure that your manuscript meets PLOS ONE's style requirements, including those for file naming. The PLOS ONE style templates can be found at

*Style requirements have been checked.*

2. Please include a complete copy of PLOS’ questionnaire on inclusivity in global research in your revised manuscript. Our policy for research in this area aims to improve transparency in the reporting of research performed outside of researchers’ own country or community. The policy applies to researchers who have travelled to a different country to conduct research, research with Indigenous populations or their lands, and research on cultural artefacts. The questionnaire can also be requested at the journal’s discretion for any other submissions, even if these conditions are not met. Please find more information on the policy and a link to download a blank copy of the questionnaire here: https://journals.plos.org/plosone/s/best-practices-in-research-reporting. Please upload a completed version of your questionnaire as Supporting Information when you resubmit your manuscript.

*This has been completed and attached to the resubmission as supporting information.*

3. We note that the grant information you provided in the ‘Funding Information’ and ‘Financial Disclosure’ sections do not match. When you resubmit, please ensure that you provide the correct grant numbers for the awards you received for your study in the ‘Funding Information’ section.

*Funding Information and Financial Disclosure sections do match now.*

4. Thank you for stating the following financial disclosure: The study is funded by a grant from the Erasmus+ Program of the European Union (grant number: IT02-KA220-HED- 000088065). Please state what role the funders took in the study. If the funders had no role, please state: "The funders had no role in study design, data collection and analysis, decision to publish, or preparation of the manuscript." If this statement is not correct you must amend it as needed. Please include this amended Role of Funder statement in your cover letter; we will change the online submission form on your behalf.

*Role of funder statement has now been added correctly to the cover letter.*

5. Thank you for stating the following in the Competing Interests section: M. O’Keeffe is supported by an Irish fellowship (UCD Ad Astra Fellowship). Please confirm that this does not alter your adherence to all PLOS ONE policies on sharing data and materials, by including the following statement: "This does not alter our adherence to PLOS ONE policies on sharing data and materials.” (as detailed online in our guide for authors http://journals.plos.org/plosone/s/competing-interests). If there are restrictions on sharing of data and/or materials, please state these. Please note that we cannot proceed with consideration of your article until this information has been declared. Please include your updated Competing Interests statement in your cover letter; we will change the online submission form on your behalf.

*Conflict of interest statement has been updated to include “This does not alter our adherence to PLOS ONE policies on sharing data and materials.”. We have also moved this, as requested, to the cover letter document.*

6. Please amend the manuscript submission data (via Edit Submission) to include author Nathan Skidmore and Antonio Portolés

*Manuscript has been updated to include both Nathan Skidmore and Antonio Portolés as authors.*

7. Please amend your authorship list in your manuscript file to include author Nathaniel Skidmore

*Authorship list has been corrected to include Nathan Skidmore consistently throughout all documents.*

8. One of the noted authors is a group or consortium PANACEA Consortium. In addition to naming the author group, please list the individual authors and affiliations within this group in the acknowledgments section of your manuscript. Please also indicate clearly a lead author for this group along with a contact email address.

*Members of the PANACEA consortium have now been added to the acknowledgement section of the manuscript.*

9. Please include captions for your Supporting Information files at the end of your manuscript, and update any in-text citations to match accordingly. Please see our Supporting Information guidelines for more information: http://journals.plos.org/plosone/s/supporting-information.

*Captions for S1-S4 have been added at the end of the manuscript.*

10. Please remove all personal information, ensure that the data shared are in accordance with participant consent, and re-upload a fully anonymized data set. Note: spreadsheet columns with personal information must be removed and not hidden as all hidden columns will appear in the published file. Additional guidance on preparing raw data for publication can be found in our Data Policy (https://journals.plos.org/plosone/s/data-availability#loc-human-research-participant-data-and-other-sensitive-data) and in the following article: http://www.bmj.com/content/340/bmj.c181.long.

*Thank you for this important reminder. We did not upload any dataset. However, we have reviewed the manuscript and supplementary materials and confirmed that no personal identifiers (e.g., names, email addresses, IP addresses) are included.*

11. If the reviewer comments include a recommendation to cite specific previously published works, please review and evaluate these publications to determine whether they are relevant and should be cited. There is no requirement to cite these works unless the editor has indicated otherwise.

*Understood. The recommendations from the reviewers have been carefully considered.*

**Reviewer #1**

“This manuscript is clearly written and demonstrates a rigorously executed mixed-method study that adds depth to our understanding of healthcare professionals’ knowledge and attitudes regarding placebo and nocebo effects. Particularly, I appreciated the correct use of descriptive statistics where incorrect use of p-values is widespread. While the study is promising and contributes valuable insights to the field, several minor revisions are needed to enhance clarity, accuracy, and consistency throughout the manuscript. I have also provided multiple suggestions for clarification and improvement. Particularly, the introduction would benefit from using more accurate references and deepening the rationale for the study’s contribution. The results and discussion contain several ambiguities or inconsistencies that need to be addressed. In the discussion, overgeneralization from qualitative findings should be avoided, and several limitations should be acknowledged due to the sample size and composition.”

*We thank the reviewer for their exceptional detail in the review of our manuscript. The detail you have provided provides us with an opportunity to be succinct and targeted in our modifications to improve the manuscript. Below we have addressed each comment individually with responses provided, in the order that the comments were provided.*

1. OVERALL: One thought that may be considered is to substitute the use of “the placebo effect” with “placebo effects” when appropriate. Because there are various mechanisms behind placebo effects and several levers to activate these mechanisms, it may be more accurate to evoke placebo effects in a plural sense. 1 Up to the authors.

*We thank the reviewer for this thoughtful comment. We agree that using the plural form “placebo effects” is often more accurate to reflect the variety of underlying mechanisms, and we have revised the manuscript accordingly where appropriate.*

2. INTRODUCTION: I believe a bibliometric reference would be better suited to support the claim on line 70 instead of references 4 & 5. Maybe something such as: Weimer, K., Buschhart, C., Broelz, E. K., Enck, P., & Horing, B. (2022). Bibliometric properties of placebo literature from the JIPS database: A descriptive study. Frontiers in Psychiatry, 13, 853953. https://doi.org/10.3389/fpsyt.2022.853953

*This passage (lines 64-70) now reads “Placebo effects concern the changes specifically attributable to placebo mechanisms. That is, the neurobiological and psychological mechanisms arising from patients experiencing symptom improvements purely from the expectation of care, the therapeutic environment, or interactions with healthcare providers. All these can significantly influence a patient’s perception of health and recovery. Conversely, nocebo effects entail negative health changes driven by psychological and biological mechanisms, activated by individual and contextual factors (1-4).*

3. INTRODUCTION: There may be some claims that the use of contextual factors does not produce clinically meaningful effects on their own. See: o Saueressig, T., Owen, P. J., Pedder, H., Arora, N. K., Simons, M., Kaczorowski, S., Miller, C. T., Donath, L., & Belavy, D. L. (2024). Boosting treatment outcomes via the patient-practitioner relationship, treatment-beliefs or therapeutic setting. A systematic review with meta-analysis of contextual effects in chronic musculoskeletal pain. The Journal of Orthopaedic and Sports Physical Therapy, 1–51. https://doi.org/10.2519/jospt.2024.12259

*Thank you for this comment. To acknowledge the existence of different perspectives in the field, we propose to add a short note in the introduction which refers to recent work questioning the extent of these effects. We believe this solution allows us to remain accurate while fairly representing the scientific debate.*

*Lines 78-81:*

*“While the existing literature provides robust evidence supporting a significant influence of healthcare professional interactions on outcomes, it is important to acknowledge that some recent works, highlight ongoing debate about the precise role and impact of the healthcare provider in this process (14).”*

4. INTRODUCTION: I think the sentence on line 81-82 may need to be clarified.

*Thank you for your suggestion. We have revised the readability of this section and believe is now suitable (see next comment).*

5. INTRODUCTION: I’m not sure whether the question (L82) remains whether deceptive treatments can be administered ethically, but rather how placebo treatments may be used without infringing upon autonomy. To this end, open-label placebos have been a topic of growing interest. This is all the more relevant given that there are studies that have either found no superiority of a deceptive placebo over an open-label placebo2, and one other that has demonstrated non-inferiority3. Nonetheless, deceptive placebo treatments are still largely used in practice.

*We thank the reviewer for these comments (4-5). We have now clarified better the sentence (lines 85-97):*

*“Additionally, the lack of clarity in defining and applying the terms ‘placebo’ and ‘placebo effects’ raises important ethical considerations. The debate has shifted from questioning whether it is ethically acceptable to prescribe deceptive placebo treatment, to exploring how placebo interventions may be used without infringing on patient autonomy. By "placebo intervention," we generally refer to an approach that encompasses both tangible (such as pills or procedures) and contextual (such as the therapeutic environment and provider-patient interaction) factors that activate placebo effects, influencing patient responses through expectations and environmental cues. In this context, open-label placebos (i.e., honestly prescribed placebo interventions) have attracted growing interest, as several studies have shown either no superiority of deceptive placebos over open-label ones, or even non-inferiority. Nevertheless, deceptive placebo treatments remain common in clinical practice (17, 18). Healthcare professionals often navigate these ethical challenges while deliberately aiming to induce and manage placebo and nocebo effects based largely on personal experiences rather than sound scientific evidence (17, 19).”*

6. INTRODUCTION: References 14 & 16 may not be the most suitable for the claim of line 86, given that these references were about placebo treatments and not implementing placebo effects in a larger sense (i.e., through the use of placebo treatments or the use of contextual factors).

*We agree that the original references focused more on placebo treatments rather than broader placebo/nocebo effects and their contextual implementation. We have retained the original reference (Linde et al., 2018) to reflect physician-reported practices regarding impure placebo use, and have now added Druart et al. (2023) and Locher et al. (2017) to strengthen this point by incorporating evidence on the efficacy and ethical considerations of open-label placebo interventions, as recommended.*

7. INTRODUCTION: The references on line 98 mainly discuss acceptability from a physician’s perspective. It could be worth adding one reference about the efficacy of 1 “As emphasized in this review, we now know that there is not a single but many placebo effects, with different mechanisms and in different diseases, systems, and therapeutic interventions.”

*Thank you for this helpful suggestion. We have retained the original reference (Linde et al., 2018) to reflect physician-reported practices regarding impure placebo use and have now added Druart et al. (2023) and Locher et al. (2017) to strengthen this point by incorporating evidence on the efficacy and ethical considerations of open-label placebo interventions, as recommended.*

8. INTRODUCTION: On line 108, reference 22 should be mentioned. In that study, the population was exactly the target population mentioned: European healthcare professionals (HCP). Additionally, what difference does it make to have a survey for all HCPs? There are several surveys on physicians, medical students, nurses, nursing students, pharmacists, physiotherapists, and physiotherapy students. This might be worth expanding on in the following revision.

*Thank you for this helpful suggestion. We have added Druart et al. (2023) to support the claim regarding variability in the definition and use of placebo interventions, particularly as this study directly surveyed European healthcare professionals. We agree this reference strengthens the rationale for further investigation into the motivations behind placebo use in real-world clinical settings (line 111). Please also refer to our reply to comment #9.*

9. INTRODUCTION: The introduction could be strengthened (L106 onwards) with a greater emphasis on how this study contributes to the existing literature. As it stands, I’m unsure what this study brings to the existing body of literature. The main originality seems to be its focus on including nocebo effects and providing more in-depth information about the training HCPs might wish for.

*We have revised the final paragraph of the introduction to clarify the unique contributions of our study. Specifically, its inclusion of a broader range of healthcare professionals, its focus on both placebo and nocebo effects, and its attention to clinicians’ training preferences. These elements offer new insights that extend beyond existing literature and support the development of practical, ethically grounded educational resources. We have also been conscious of the length of the introduction and have made efforts to make this part as succinct as possible.*

*Lines: 124-132:*

*“This study builds on existing research by including a broader range of healthcare professionals, including medical professionals (e.g., family physicians, anesthesiologists, surgeons, neurologists) and nurses, as well as physiotherapists and psychologists. Moreover, this study extends prior research by not only considering placebo effects but also explicitly addressing nocebo effects, an area that has received comparatively little attention. Moreover, it goes beyond mapping knowledge and attitudes by also providing in-depth insights into healthcare professionals’ preferences for training, thereby offering concrete directions for the design and implementation of future educational programs. The findings will inform the development of educational resources to ensure the ethical and effective integration of placebo and nocebo effects into clinical practice.”*

10. METHODS: How was the survey developed (L127) regarding previous surveys? Were there sections that were taken verbatim? COSMIN recommendations for the use of measurement instruments are first to use existing ones and adapt only if necessary.

*We thank the reviewer for this important comment. We recognize the importance of adhering to COSMIN recommendations, which prioritize the use of validated measurement instruments when available. Given the lack of validated surveys specifically addressing the combination of issues we aimed to investigate, we conducted a thorough literature review to identify existing questionnaires on related topics. We extracted and examined these surveys to assess their relevance, consistency of items with our study aims, and reported psychometric properties (when available).*

*Based on this review, we selected items from existing instruments that demonstrated relevance and psychometric validity, and integrated them with additional closed and open-ended questions designed to address the specific objectives of our study. The draft survey was then reviewed by external experts to evaluate clarity, ease of understanding, and estimated completion time, ensuring content validity and feasibility.*

*In this way, our approach was guided by COSMIN principles: we prioritized existing validated items where available and adapted content only when necessary to meet our specific research goals, ensuring a rigorous and transparent development process.*

*We have now revised the manuscript to clarify the development process of our survey, explicitly explaining how we prioritized existing instruments, assessed their relevance and psychometric properties, and adapted items in accordance with COSMIN principles.*

*Lines 138-143:*

*“The survey was developed in accordance with COSMIN principles, which prioritize the use of validated instruments where available. We conducted a structured review of existing questionnaires on related topics (15, 17, 24-29), to identify relevant items and evaluate their alignment with our study objectives. Items judged to be relevant and psychometrically sound were incorporated, and additional close and open-ended questions were developed to address gaps not covered by existing tools.”*

11. METHODS: Given the diverse population (L138), in how many languages was the questionnaire developed? How were the translations done? If only in English, I believe this would be a limitation of the study that should be mentioned. / It seems the answer comes on line 150.

*Correct, the survey was only developed in English language. This has now been acknowledged as a limitation appropriately. The manuscript now reads “Finally, alongside a reliance on self-reported data introducing a potential response bias, the survey was provided in English language only limiting responses from non-English speakers.” (Lines 508-510).*

12. METHODS: If the study is carried out in multiple different countries, would the ethical approval from an Italian ethics committee suffice? (L132) Would it be necessary to obtain ethical approval for each country? I don’t think this is the case, but do the authors have any knowledge of this?

*This was a discussion within the early stages of the research design. It is concluded that ethical approval from the named institution is sufficient in this case.*

13. METHODS: How was healthcare professional defined in this context? (L138) From experience, there are different interpretations in the population about who is included as a HCP (e.g., osteopaths, chiropractors?), and variations exist among countries (e.g., psychologists). Were there any exclusion criteria?

*Thank you for your comments. In this study, the classification of "healthcare professional" was based on self-reported professional identity. We did not apply exclusion criteria based on professional background, as we recognised and sought to accommodate the diversity of healthcare roles across European contexts. This inclusive approach aligns with the study’s objective to explore a broad spectrum of perspectives on placebo and nocebo effects among practicing professionals. We have now specified it in the manuscript.*

*Line 154-155:*

*“Qualified healthcare professionals (self-reported) actively working in Europe from any healthcare discipline or specialty were included.”*

14. METHODS: How do the authors explain the high rate of partially completed surveys? (L184) How far did the participants get into the survey before dropping out? This may be due to the questionnaire being too long. Recommendations are often to keep the questionnaire to a maximum of 7 minutes. What is the rationale for including the incomplete answers? How far did they go into the questionnaire? Also, I’m not sure I understand the fully and partially N=604 and N=807 figures. At the moment, I deduce that the 807 is the total number of consents, of which only 212 were complete and 604 were incomplete. So the N refers to the total number of questionnaires in one case and the number of incomplete questionnaires in another. Please clarify.

*Thank you for raising these important points. To clarify: a total of 807 respondents consented and began the survey. Of these, 203 (25.2%) completed all items, while 604 (74.8%) provided partial responses. The originally reported numbers may have caused confusion, and we have revised the Methods and Results sections to clearly state the total sample and the split between full and partial completions.*

*The high rate of partial completion is most likely explained by the nature of the survey design. The questionnaire included 46 items across multiple domains and incorporated both closed- and open-ended questions. Although the intended completion time was approximately 15 minutes, the observed average completion time among those who finished was around 30 minutes. This duration is longer than the 7–10 minute window often recommended for maximising completion rates in online survey as you’ve mentioned. In addition, the survey was open-access, voluntary, and uncompensated; participants could exit at any point without penalty. Taken together, these features likely contributed to the attrition observed.*

*We chose to include partial responses in our analyses because many of these participants completed substantial portions of the survey, and their data remain valid for the items they answered. Excluding them would have unnecessarily reduced the sample size and may have introduced bias by restricting analyses to only those who had the time and motivation to complete the full instrument. In accordance with the CHERRIES checklist, we report both complete and partial response rates transparently and clearly describe their inclusion in analyses.*

*We also examined the distribution of dropouts across the questionnaire. While attrition increased gradually across the survey length, there was no evidence of systematic dropout clustered in a specific section (e.g., ethics or training), suggesting that partial completions reflected respondent burden rather than avoidance of particular topics. We agree that the high rate of partial completion represents a limitation for the study, and we have explicitly acknowledged this in the Limitations section. However, we consider that inclusion of partial responses provides a more complete and representative account of healthcare professionals’ perspectives than restricting to only the fully completed questionnaires.*

*Lines 208-211:*

*“Both complete and partial responses were included in the analyses. Responses were considered complete if all items of the survey were answered and partial if only a subset of items were completed. Partial responses were retained, as many participants completed substantial sections of the survey, and their data were considered valid for the items they answered.”*

*Lines 510-513:*

*“The high proportion of partial completions also represents a limitation. Nevertheless, these responses were included in analyses because many participants completed substantial sections of the survey, and their data were considered valid for the items answered.”*

15. METHODS: I appreciate the appropriate use of descriptive statistics (L185) where the conditions for testing under H0 are not present. This is all the more worth highlighting given the pressure often put on researchers to produce p-values.

*We thank the reviewer for this positive comment. We fully agree that descriptive statistics were the most appropriate choice in this case, given that the conditions for testing under the null hypothesis were not met. We also appreciate the acknowledgment of our decision to avoid reporting p-values where inferential analyses would not have been justified.*

16. METHODS: Please provide a brief description of the profiles of the authors involved in the qualitative analysis (as suggested by the COREQ items). L193 onwards

*Thank you for your suggestion. While we acknowledge that the COREQ guidelines are primarily designed for qualitative studies based on interviews or focus groups, and therefore may not fully apply to this survey-based analysis of open-text responses, we agree that providing brief information on the backgrounds of the authors involved in the qualitative analysis can enhance transparency. Accordingly, we have included a description of their relevant expertise and roles in the revised manuscript.*

*Lines 227-230:*

*“NS, a musculoskeletal therapist (PhD, male), and MOK, a physiotherapist (PhD, female) both with experience in qualitative research with no previously established direct relationships with the respondents, conducted the qualitative component of this study.”*

17. METHODS: On line 204, why was the choice made to draw the framework from a theory-driven manner and not a data-driven one?

*Coding frameworks were developed using a bottom-up approach derived from the data, which informed the identification of appropriate representative categories. Consequently, we do not assume a dichotomous interpretation of the data.*

18. METHODS: Were the other authors then included in the qualitative analysis or was it solely the work of MOK and NS? L225

*Only MOK and NS were involved in the qualitative analysis. To strengthen the rigor of the process, their work was subsequently re-evaluated independently and blinded by two additional authors, KM and AB.*

*Lines 252-253:*

*“To strengthen the rigor of the process, MOK and NS work was subsequently re-evaluated independently and blinded by two additional authors, KM and AB.”*

19. RESULTS: Was demographic data not included as a condition for the questionnaire to count as complete? If not, why was this choice made? Were the questions required or skippable? This is a very low portion of the respondents.

*Participants were permitted to omit responses to certain questions, a strategy designed to enhance respondent participation and adhere to guidelines advocating for voluntary engagement, including the option to refrain from answering specific questions. Demographic data was collected in a dedicated section at the end of the survey, because this information was less central to the study objectives compared to the questions directly related to the research aims. This placement also aimed to give priority to items more closely aligned with the main subject of the study, increasing the likelihood that respondents would complete the most relevant questions. While participants were encouraged to complete all questions, this approach was intended to mitigate data loss for key variables if respondents chose to terminate the survey early. We have now clarified this approach in the main manuscript.*

*Lines 172-178:*

*“The demographic data was collected in a dedicated section at the end of the survey, because this information was less central to the study objectives compared to the questions directly related to the research aims. This placement also aimed to give priority to items closely aligned with the main subject of the study, increasing the likelihood that respondents would complete the most relevant questions. While participants were encouraged to complete all questions, this approach was intended to mitigate data loss for key variables if respondents chose to terminate the survey early.”*

20. RESULTS: L244, with such a broad population base, the participation is very low. Several large countries were sparsely represented. This should be discussed as a limitation of the study’s extrapolation to a larger population. The main participating countries are those of the most represented affiliation of authors. Perhaps in future studies, the PANACEA consortium should aim to establish a more extensive network throughout Europe. Notably, it is missing people/responses from Germany, the United Kingdom, France, and Spain, to name a few of the larger countries with active placebo researchers, in order of population.

*We fully agree that this is a very good point.* *Text has been added in the manuscript to acknowledge this: “With such a broad potential population base, overall participation was low, and several large countries, with active placebo researchers and large populations, were sparsely represented. This underrepresentation limits the extrapolation of findings to a wider European context and reflects the tendency for main participating countries to align with the affiliations of study authors.*

*Lines 502-506:*

*“Considering the overrepresentation of Italian participants (55.19%), and pain professionals (31.6%) and such a broad potential population base, overall participation was low, and several large countries, with active placebo researchers and large populations, were sparsely represented. This underrepresentation limits the extrapolation of findings to a wider European context and reflects the tendency for main participating countries to align with the affiliations of study authors.”*

21. FIGURE 1: What does the Other category comprise? This links to comment n°13

*Thank you for this valuable observation. In our study, healthcare professional status was self-reported by participants, and we did not apply exclusion criteria based on specific professional titles. This was a deliberate decision to reflect the diversity of healthcare roles across European countries, a decision which resulted in a highly varied group of healthcare professionals with some specialties being represented only by a few members. To provide greater transparency, we have now added a summary of the reported specialties to the results section.*

*Lines 279-285:*

*“Within the self-reported ‘Other specialty’ category (n=57, 17%), a wide range of medical specialties were provided. The most frequently reported were gynecology (n=10) and emergency internal medicine (n=7) and pain medicine (n = 5), followed by internal medicine (n=5), clinical pharmacology and physiotherapy (n = 4). Emergency medicine and intensive care were each reported by three respondents. Specialties reported by two participants included anesthesiology and geriatrics. A wide variety of single-response other specialties were also recorded, such as dentistry, hematology, endocrinology, nephrology, otorhinolaryngology, midwifery, and palliative care.”*

22. RESULTS: L254. Was there any mention of the other components of the placebo response apart from placebo effects?

*Thank you for this question. We believe the reviewer is asking whether any of the respondents referred to changes in patient health outcomes not attributable to an active treatment or placebo mechanisms, but rather to other, unrelated phenomena, such as regression to the mean or natural disease progression. Table 1 captures perceived contributors to the placebo effect, including natural disease progression, regression to the mean etc.*

23. RESULTS: I highly appreciate the direct inclusion of participant quotes in this presentation of the mixed data. It might be necessary to mention what the number is in quotes. I’m guessing it’s the participant’s age.

*“Age” has now been added as an indicator to each comment provided throughout the manuscript.*

24. TABLE 1: I’m not clear on the options chosen for question 12. Are these questions about the contextual factors that trigger placebo or nocebo effects? If not, maybe consider switching from the word factor used in the question above to limit confusion. Additionally, an item such as “A treatment with an active ingredient” might have helped investigate whether participants understood that placebo responses and effects occur in every healthcare interaction.

*To avoid confusion, we have revised the label in Table 1 and the corresponding section of the results to read “Perceived contributors to the placebo effect,” reflecting that these items represent participant beliefs rather than established causal factors. We also acknowledge the value of including an item on “treatment with an active ingredient” in future surveys to assess broader understandings of placebo responses, including how such effects can occur in routine clinical interactions and not just with inert or non-specific interventions. Thank you for that insight.*

25. RESULTS: I think the sentence line 308 should be rephrased, as conditions could also be a synonym of ailment or pathology. Here, maybe specialities, disciplines, or fields may be better suited.

*“Clinical disciplines” is now the term used in this case. Thank you for your feedback, this now reads much more clearly.*

26. TABLE 2: Why was it chosen to use different treatment instead of different forms of treatment? For instance, is it thought that pills have different placebo responses depending on whether they are given to someone with depression or anxiety? Wouldn’t this be rather a confound with what conditions people think are susceptible to placebo responses, or even whether the treatments have a large specific effect, or if their effect is mainly a placebo response?

*We thank the reviewer for this thoughtful comment. Our intention in formulating the survey item was not to compare different forms of treatment (e.g., pills vs injections), but rather to explore participants’ perceptions of how specific treatments, as they are typically encountered in clinical contexts, may contribute to activating placebo effects. We acknowledge that this approach can introduce confounding with factors such as the condition being treated, perceived susceptibility to placebo responses, or the balance between specific and nonspecific effects. However, we considered this trade-off acceptable, as our aim was to capture respondents’ intuitive beliefs about real-world treatments rather than abstract treatment modalities.*

27. TABLE 2: In Table 1, the name of each subitem was given, and they are not in Table 2. Please pick one convention in reporting results.

*In table 1 each subitem is provided. This has now been changed, given the lack of reference to the subitems in the remainder of the manuscript, to show simply “Factors contributing to the placebo effect (Item 12) 237/807 (29.4%)”. This is now reported consistently and conventionally with the remainder of the tables. Moreover, we have now adopted a consistent convention: in Table 1 we report only the item number in parentheses (e.g., item 12) when it corresponds to a question, and we removed references to the response options (e.g., item 12a).*

28. TABLE 2: Were the placebo treatments described in the question? If the placebo treatment is pure or impure, deceptive or open, this may contextualize the responses.

*Thank you for your thoughtful observation. We agree that there is an important distinction between administering placebo treatments and enhancing placebo effects during routine care. While this distinction is clearly framed in Table 2, we have now revised the accompanying text to reflect this more explicitly. Our intention was to avoid duplicating table content, but we appreciate the need for clarity in the narrative and have added wording to ensure the reader understands that the focus of this section is on clinical strategies that aim to enhance placebo effects rather than the use of inert treatments.*

29. RESULTS: This section should clarify whether it is referring to placebo treatments or enhancing placebo effects in daily care. This is correctly done in the table. On a side note, using placebo effects without consent could also occur without the knowledge of providers. In a way, HCPs who have little to no knowledge about placebo effects may still be promoting them intuitively. Additionally, I believe the second part of Table 2 is pertinent to the ethics of using placebo treatments and their effects.

*We agree that it is important to clarify the distinction between placebo treatments and the broader activation of placebo effects in clinical care. We have revised the relevant section of the results to clarify this distinction (line 361) and to highlight that the second part of Table 2 relates to ethical considerations in both intentional and unintentional use of placebo mechanisms. Additionally, we have acknowledged in the discussion that placebo effects may be activated intuitively or without provider awareness, which reinforces the importance of foundational training for all healthcare professionals.*

*Lines 478-481:*

*“In addition, placebo and nocebo responses may be triggered unintentionally through routine communication and care, even without provider awareness. This underscores the importance of ensuring all healthcare professionals receive foundational training to recognize and manage these effects.”*

30. TABLE 5: I think the title for Table 5 could be made more explicit. The text suggests that this was the result of asking how participants would like to learn about placebo and nocebo effects. The title of the table indicates that these are the preferred methods of learning for HCPs.

*Title has now been changed to “Perceived helpfulness of previous training methods” to provide better detail.*

31. DISCUSSION: I’m not too fond of the term 'placebo intervention,’ which may refer to a non-pharmacological placebo treatment (or placebo intervention), whereas I believe it was meant to say: high prevalence of the use of placebo treatments or effects. As the authors stated in the introduction, there is a need for crystal clear clarity on these terms and notions, thus my nitpicking.

*Thank you for your valuable comment and for emphasizing the importance of using precise and clear terminology. While we have retained the term "intervention" as a broad concept encompassing all factors that can activate a placebo effect in healthcare, your suggestion prompted us to clarify this notion further.*

*Therefore, we have revised the text in the Introduction to include the following definition (lines 88-92): “By "placebo intervention," we refer to an approach that encompasses both tangible (such as pills or procedures) and contextual (such as the therapeutic environment and provider-patient interaction) factors that activate placebo effects, influencing patient responses through expectations and environmental cues.*

32. DISCUSSION: I think “specialities” is more appropriate here (L399).

*Thank you. The passage now reads (lines 418 – 420):*

*“Medical professionals echoed these concerns, emphasizing that ethical considerations must accompany practical applications in areas like oncology, surgery, and pain management.”*

33. DISCUSSION: This finding of HCPs underestimating their role is similar to what was found on reference 22.

*We have now explicitly acknowledged (line 455) the similarity between our findings and those of Druart et al. (2023), highlighting how our data also suggest that healthcare professionals may underestimate their role in eliciting placebo effects. This comparison has been added to the discussion section to strengthen the interpretation of our findings within the context of existing literature. This strengthens the arguments made, thank you for this suggestion.*

34. DISCUSSION: To my understanding, only placebo effect use was measured in this study (Question 13). Could you please check the use of 'placebo treatment' instead of 'effect' in this sentence, please?

*Thank you for flagging this. You are correct. Question 13 specifically asked about the use of placebo effects, not placebo treatments. We have reviewed and amended the relevant sentence in the discussion to ensure the terminology accurately reflects the survey item.*

*Lines 453-454:*

*“While many reported taking advantage of placebo effects, the understanding of the underlying mechanisms varied significantly across disciplines.”*

35. DISCUSSION: I’m not sure the data here supports an inter-group cross-profession generalization (L411). It seems an over-simplification of rich qualitative data at first glance. At best, the qualitative data allows for the generation of the hypothesis that understanding varies across disciplines. These differences could also be due to age or area of practice. However, the analysis chose to look at profession, which could be the explanatory factor for these differences.

*Thank you for this thoughtful observation. We agree that while our qualitative findings captured genuine differences within our sample, these should not be over-interpreted as definitive inter-professional generalisations. We have revised the Limitations section to clarify that the transferability of these findings to the broader European healthcare workforce should be considered with caution, as other factors (e.g., age or clinical setting) may also play a role.*

*Lines 513-515:*

*“In addition, while the findings captured genuine insights within our sample, their transferability across the broader European healthcare workforce should be considered appropriately, as other factors (e.g., age, clinical setting) may also play a role.”*

36. DISCUSSION: As a side note, it would be interesting to see if they felt more confident than others in harnessing placebo effects, given that they were prescribed as impure placebos by other professionals. L422

*Our analysis did not examine confidence levels in relation to whether respondents reported prescribing interventions considered impure placebos. Exploring whether prescribing impure placebos is associated with greater confidence in harnessing placebo effects is beyond the scope of the present analysis, but we agree it would be an important avenue for future research.*

37. DISCUSSION: Was the lack of training mediated by years since graduation? This might help understand whether training is more implemented in initial health training or not. L439

*Thank you for this insightful comment. We did collect information on years of clinical experience (see Figure 2), which provides a proxy for time since qualification. However, we did not analyse training status in relation to this variable, and our survey did not specifically distinguish between training received during initial qualification versus post-qualification continuing professional development. The data we do have show that the majority of respondents reported receiving no formal training on placebo (63.1%) or nocebo effects (67.7%). This suggests that, at least within our sample, structured education on these topics is not yet widely embedded across professional trajectories. While it is possible that more recent graduates may have had greater exposure through evolving curricula, we were unable to confirm this with our dataset. We agree that examining whether training is more commonly delivered during initial qualification or later in professional careers would provide valuable insight, and we have highlighted this as an important area for future research.*

*Lines 493-495:*

*“Future research should examine whether training is introduced during initial qualifications or later in professional development, to inform how best to embed these topics across career stages.”*

38. DISCUSSION: These seem like findings rather than discussion points. They are probably the most relevant finding for this section of the survey. L447

*Thank you for this helpful comment. We agree and have revised this section of the Discussion to reduce repetition of specific results and instead emphasise their interpretation and implications. The text now highlights the gap between awareness and application, and frames this in terms of the need for targeted education on placebo and nocebo effects, rather than restating numerical findings.*

*Lines 466-469:*

*“Respondents reported generally low confidence in harnessing placebo effects and preventing nocebo effects, highlighting a clear gap between awareness of these phenomena and the ability to apply them effectively in practice. This gap underscores the importance of targeted education focusing on practical strategies, ethical considerations, and communication skills.”*

39. DISCUSSION: The over-representation of pain professionals should be expected given the recruitment through the European Pain Federation EFIC. In addition to previous comments on the participants, it should be noted not only the overrepresentation but also the underrepresentation of specific demographics, as stated above. Thus, the first strength may be overstated.

*We have revised the strengths paragraph to more cautiously frame the contribution of the international sample. The text now acknowledges that, while inclusion of participants from multiple countries broadens the scope beyond single-nation studies, uneven representation across countries and professions should temper the generalizability of the findings.*

*Lines 502-506:*

*“Considering the overrepresentation of Italian participants (55.19%), and pain professionals (31.6%) and such a broad potential population base, overall participation was low, and several large countries, with active placebo researchers and large populations, were sparsely represented. This underrepresentation limits the extrapolation of findings to a wider European context and reflects the tendency for main participating countries to align with the affiliations of study authors.”*

40. DISCUSSION: I believe the ethicality could have benefited from a qualitative section as well. As it stands, I’m unsure it’s more detailed than other surveys. In my opinion, the use of mixed methods here is the key strength of this project, and it allowed for an indepth understanding of placebo knowledge that was lacking in several previous studies on the topic

*Thank you for this thoughtful feedback. We agree that a qualitative section dedicated specifically to ethicality could have provided additional depth, and we recognise this as a valuable avenue for future research. At the same time, we would emphasise that our mixed-methods design was a deliberate choice to capture a richer and more nuanced understanding of healthcare professionals’ perspectives than has been reported in previous surveys. We are pleased that you highlighted this aspect as a key strength.*

**Reviewer #2**

“The current manuscript examined health care professionals’ knowledge on placebo and nocebo effects by means of a survey. I enjoyed reading the manuscript. Please see below for some minor comments.”

*We thank the reviewer for the in-depth comments. We have made several modifications to the manuscript to improve the below points and overall readability and grammar. Please see below the changes made in detail.*

**Introduction**

1. Clear introduction, written to the point. Since it is a short come of previous studies, could be it an example of ‘diverse range of healthcare professionals’ be given in the last paragraph of the introduction?

*Thank you for this observation. We have clarified the contribution of our study in the revised final paragraph of the introduction, highlighting the inclusion of a broader range of healthcare professionals and giving some examples.*

*Lines 124-126:*

*“This study builds on existing research by including a broader range of healthcare professionals, including medical professionals (e.g., family physicians, anesthesiologists, surgeons, neurologists) and nurses, as well as physiotherapists and psychologists.”*

**Method**

2. Recruitment: is there a flow chain of number of participants were approached, responded etc. to present a response rate? Were reminders sent?

*Thank you for this comment. As this was an open, web-based survey distributed through targeted communication channels (website, newsletter, social media) and partner mailing lists, we are unable to provide an exact denominator for how many individuals were approached, and therefore cannot calculate a formal response rate. No targeted reminders were sent, though the survey link remained accessible throughout the recruitment period and was promoted once in each newsletter and across social media channels. This recruitment approach was considered most appropriate for reaching a diverse, international population of healthcare professionals across multiple disciplines, and to capture perspectives beyond those available through closed professional networks or single-country sampling. This is clarified in the Methods section to improve transparency.*

*Lines 163-165:*

*“Because recruitment was open through these channels, the number of individuals reached could not be determined, and therefore the formal response rate was not calculable.”*

3. It is clear that the order of questions was not randomized, but were the topics counterbalanced?

*Thank you for this question. The topics were not counterbalanced. This was a deliberate choice, as the survey was structured to first capture respondents’ unprompted understanding of placebo and nocebo phenomena before introducing these terms. Counterbalancing or reordering items may have risked priming participants and reducing the validity of this baseline assessment. We have now specified that topics were also not counterbalanced.*

*Lines 168-170:*

*“The order of the questions was not randomized, and the topics were not counterbalanced, as the initial section aimed to gather participants' understanding of the placebo effects without explicitly using the term ‘placebo’.”*

4. How much time, on average, did participants need to complete the survey?

*Thank you for this comment. Among the 203 respondents who completed 100% of the survey, the average completion time was 30 minutes and 7 seconds. We have added this detail to the Results section.*

*Line 271-273:*

*“Among the 203 respondents who fully completed the survey, the average completion time was 30 minutes and seven seconds.”*

5. Were there any control items? If so, how many, and how many participants failed the control questions? And was their data omitted?

*Thank you for this question. No formal control items (e.g., attention checks) were included in the survey. This decision was made to minimise burden on respondents and avoid reducing completion rates. Instead, data quality was safeguarded through design features such as IP address marking (to prevent duplicate entries) and by including both fully (25.2%, n=203 and partially (74.8%, n=604) completed responses in the analysis. No participants were excluded on the basis of control questions, as none were used. We have specified the no control items were included in the survey.*

*Lines 179-180:*

*“No formal control items (e.g., attention checks) were included in the survey to minimize respondent burden;”*

**Results**

6. Could numbers be listed next to percentages?

*As requested, we have added the corresponding numbers alongside the percentages in the Results section.*

7. Could figure 2 be transformed to a table? It seems easier to read the information there. And perhaps add the numbers of discipline from Figure 1 as well.

*Thank you for this suggestion. We considered presenting Figure 2 as a table in our original draft; however, given the number of tables already included in the manuscript, we felt that retaining this information as a figure provided a clearer balance between text, tables, and figures. We agree that clarity is important, so we have ensured the figure and its legend are fully detailed.*

8. Very interesting results in Figure 3 – was it also explained why the listed disciplines were believed to be most prone to placebo and nocebo effects?

*Thank you for this comment. The survey captured which disciplines were perceived as most influenced by placebo and nocebo effects, but it did not include follow-up questions asking participants to explain why they considered these disciplines more prone. We agree that exploring the reasoning behind these perceptions would provide valuable additional insight and represents an important direction for future qualitative research.*

**Results/Discussion**

9. The results are added together – also perhaps due to the smaller sample sizes e.g., in psychologists and nurses. However, although the placebo and nocebo effects are psychological effects, the treatment provided by the professionals are different. That is, a side effect due to medicine might be experienced differently (e.g., ascribed to the pill) than to another person (e.g., a psychologist). I think this difference in treatment type might also influence results and interpretations of placebo/nocebo. In psychology, it is not clear what nocebo effects are, since therapeutic side effects is an understudied topic. This is discussed later on in the discussion, but if professionals do not have the knowledge about nocebo effects int heir own field, it is perhaps hard to conclude there is a knowledge gap when there is no knowledge to begin with, since e.g., RCTs do not take nocebo effects into account.

*Thank you for this thoughtful discussion point, very much in line with our research. We agree that differences in treatment context across professions (e.g., pharmacological versus psychological interventions) are likely to shape how placebo and nocebo effects are understood and experienced. As you note, therapeutic side effects are an under-studied area in psychology, which makes interpretation of nocebo effects in this discipline particularly challenging. Because of smaller subgroup sizes, we reported results in aggregate to allow for meaningful quantitative analysis, but we have clarified that variation in treatment type and disciplinary context should be considered when interpreting these findings. We also agree that in some areas it may be more accurate to describe the issue as a lack of foundational knowledge rather than a “knowledge gap,” and we have nuanced our wording accordingly. We have recognized this aspect in our Limitations.*

*Lines 515-519:*

*“Finally, it should be acknowledged that the type of treatment delivered by different healthcare professionals may shape how placebo and nocebo effects are perceived and manifested. For example, side effects ascribed to a pharmacological treatment may be experienced differently than those emerging in psychological interventions. This disciplinary variability highlights the complexity of interpreting our findings across professional groups.”*

**Writing**

10. At times passive writing (e.g., Research indicates that Y) – usually everything before ‘that’ can be deleted without losing the meaning of the sentence.

*We reviewed the manuscript and revised several instances of passive or indirect phrasing to improve clarity and concision.*

11. Method: n should be italic.

*Thank you for this notice. This has now been modified.*

12. Numbers under ten should be written fully. Sentences starting with numbers should be written fully.

*Thank you for this comment. We have reviewed the manuscript and revised the text accordingly, ensuring that numbers under ten are written out in words where appropriate and that sentences beginning with numbers are spelled out in full.*

13. Typo p. 20; comma after Table4

*Thank you for this detail. We have now removed the comma correctly.*

Additional improvements to the manuscript:

Reference list updated.
