# Supplementary material for: Placebo and nocebo in clinical practice: An online cross-sectional survey of healthcare professionals from European countries on views, practices and training needs
Source: PLoS One. 2026 Jan 14;21(1):e0338905. doi: 10.1371/journal.pone.0338905 (PMC12834211; doi:10.1371/journal.pone.0338905)
Supplement: Attachment — Submitted filename: Response to Reviewers R2.docx [file pone.0338905.s007.docx]

**Response to Reviewers**

PLOS ONE

**PONE-D-25-28687R1**

**Journal requirements**

If the reviewer comments include a recommendation to cite specific previously published works, please review and evaluate these publications to determine whether they are relevant and should be cited. There is no requirement to cite these works unless the editor has indicated otherwise.

Please review your reference list to ensure that it is complete and correct. If you have cited papers that have been retracted, please include the rationale for doing so in the manuscript text, or remove these references and replace them with relevant current references. Any changes to the reference list should be mentioned in the rebuttal letter that accompanies your revised manuscript. If you need to cite a retracted article, indicate the article’s retracted status in the References list and also include a citation and full reference for the retraction notice.

**Additional Editor Comments:**

Reviewer 1 has accepted the revised manuscript for publication, while Reviewer 3 has suggested a minor revision. Thus, I invite the authors to address the two minor but essential comments raised by Reviewer 3 and to resubmit the revised manuscript, together with the response letter, for acceptance.

**Reviewer #1**

Thank you for submitting a revised version of the manuscript and your diligence in responding to the comments. Lovely work.

*We thank the reviewer for their comment. We really appreciate it.*

**Reviewer #3**

Introductory note (for transparency):
I am joining the review process at a later stage. My comments take into account the authors’ revisions and responses since the first round. While I might not have raised exactly the same initial points as the round-one reviewers, it would be inappropriate to proceed as if starting from scratch. I therefore focus on the manuscript in its revised form, acknowledging both the improvements made and the issues that, in my view, still warrant clarification or refinement.
Against this backdrop, I now turn to my comments.

1. The study’s external validity is constrained by a convenience sample that under-represents (or does not explicitly report) major European countries, which may overstate the breadth of coverage. Although this limitation is partially addressed in the revision and responses, concerns about generalizability persist. The recruitment via specific networks, combined with the limited presence of large countries (e.g., France, Germany), may lead readers to infer a pan-European picture that the data do not fully support. To what extent can the findings be extrapolated beyond the overrepresented settings? How many respondents came from the largest European countries, and how does this distribution shape the interpretability of the descriptive estimates? Are the conclusions clearly framed as arising from a multi-country convenience sample rather than a continent-wide survey?
Given this, the title may be misleading. I recommend tempering it, e.g., “A multi-country convenience survey of European healthcare professionals on placebo/nocebo.”

*Thank you very much for this helpful comment. We fully agree that our sampling strategy limits external validity and that we need to communicate this more clearly throughout the paper, including the title. Our intention was not to present the data as a continent-wide representative survey, but rather as a multi-country convenience sample drawn from specific professional networks. We appreciate that, as currently written, the title and some formulations in the manuscript could suggest a broader level of representativeness than is warranted. To address this, we have now moderated the wording across the manuscript. See:*

*lines 1-3 (title): Placebo and Nocebo in Clinical Practice: An Online Cross-sectional Survey of Healthcare Professionals from European Countries on Views, Practices, and Training Needs*

*lines 40-41: “An online cross-sectional survey among a European multi-country convenience sample of healthcare professionals*”

*line 433: “This study examined a European multi-country convenience sample of healthcare professionals' knowledge, practices, and training needs regarding placebo and nocebo effects.”*

*In our revised manuscript, we have also emphasized that of the 807 participants who initiated the survey, only 212 completed it, which limits our demographic insights. The demographic data was collected in a dedicated section at the end of the survey because this information was less central to the study objectives compared to the questions directly related to the research aims. This placement was also intended to prioritize items closely aligned with the main subject of the study, thereby increasing the likelihood that respondents would complete the most relevant questions.*

*We have now added a paragraph in the Discussion to clarify this further.*

*See lines 504-506:*

*“Notably, out of 807 participants who initiated the survey, we have complete demographic data (last session) for only 212 respondents who fully completed the survey, limiting our ability to ascertain the precise representation of European countries within the sample.”*

2. The revised version of the manuscript substantially improves transparency and methodological reporting. To further strengthen the paper, you might consider briefly expanding the Discussion on a few residual threats to internal validity: potential order/priming effects due to a non-randomized question sequence; the absence of attention/quality checks or completion-time thresholds; possible misclassification arising from self-reported professional status; the role of incentives and recruitment channels (e.g., EFIC-linked networks) in shaping completion patterns within the sample; the lack of psychometric evidence for item clusters or composite constructs; and the absence of sensitivity analyses contrasting complete-only vs complete+partial respondents for headline estimates. A short paragraph acknowledging these points—without changing the descriptive aims—would help readers calibrate the robustness and interpretability of the findings.

*Thank you for your valuable insights. We appreciate your concerns regarding the limitations related to internal validity stemming from our sample and we have now revised the Discussion accordingly.*

*See lines 508-516*

*“Additionally, potential threats to internal validity warrant consideration. Because the sequence of questions was not randomized, we cannot exclude the possibility of order or priming effects that may have influenced participants’ responses. This design choice reflected a pragmatic decision to prioritize items most closely aligned with the main aims of the study, with the intention of maximizing completion of the most relevant questions. Furthermore, the cross-sectional design of the current study prevents the establishment of causal relationships between knowledge, attitudes, and practices. Alongside an absence of formal attention checks and a reliance on self-reported data introducing a potential response bias, the survey was provided in English language only limiting responses from non-English speakers.”*
